# Tropomyosin 1 Promotes Platelet Adhesion and Clot Contraction Separate from Its Roles in Developmental Hematopoiesis

**DOI:** 10.1002/advs.202517560

**Published:** 2026-06-04

**Authors:** Po‐Lun Kung, Victor Tsao, Alina D Peshkova, Oscar A. Marcos‐Contreras, Kim Ha, Brian M Dulmovits, Nkemdilim Okoli, Anvi Sinha, Gennadiy Fonar, Rong Qiu, Rolf D Bates, Janelle Yeboah, Carson Shalaby, Tyler Truex, Soomin Jeong, Edna C Hardeman, Peter W Gunning, Vladimir R Muzykantov, Jacob W Myerson, Christopher S Thom

**Affiliations:** ^1^ Division of Neonatology Children's Hospital of Philadelphia Philadelphia Pennsylvania USA; ^2^ Department of Pharmacology University of Pennsylvania Perelman School of Medicine Philadelphia Pennsylvania USA; ^3^ Department of Systems Pharmacology and Translational Therapeutics University of Pennsylvania Perelman School of Medicine Philadelphia Pennsylvania USA; ^4^ Department of Pathology and Laboratory Medicine Temple University Philadelphia Pennsylvania USA; ^5^ School of Biomedical Sciences University of New South Wales Sydney New South Wales Australia; ^6^ Department of Pediatrics University of Pennsylvania Perelman School of Medicine Philadelphia Pennsylvania USA

**Keywords:** actin, GWAS, hemostasis, platelet, tropomyosin 1

## Abstract

Genome‐wide association studies (GWAS) link the *Tropomyosin 1* (*Tpm1*) locus to quantitative blood trait variation, but related mechanisms are unclear. *Tpm1* encodes an actin‐binding protein that regulates actin filament diversity, cell adhesion, signaling, and actomyosin contractility. Murine *Tpm1* deficiency enhances hemogenic endothelial cell (HEC) specification, but it was unclear if these effects extended to postnatal hematopoiesis. We used *Cdh5^Cre^* and *Vav^Cre^* models to conditionally knock out *Tpm1* (*Tpm1KO*) in endothelial anor hematopoietic cells. Both models ablate *Tpm1* in postnatal blood. Endothelial *Tpm1KO* increases HEC specification without altering hematopoietic progenitor cell production or adult blood counts, suggesting separate roles for *Tpm1* in the embryonic and adult blood systems. *Tpm1KO* increases adult platelet lifespan and diminishes adhesion to fibronectin and fibrinogen. Chemical *Tpm1* inhibition also reduces focal adhesion in murine and human platelets. Altered platelet morphology and reduced platelet spreading suggest perturbed actomyosin contractility underlies these findings. Platelet fibrin binding promotes blood clot contraction, which reduces occlusive thrombosis. *Tpm1KO* limits clot contraction and worsens vascular occlusion in ferric chloride‐induced stroke models. In addition to offering a mechanistic explanation for why genetic variation at the *TPM1* alters platelet traits in GWAS, our findings reveal novel roles for *Tpm1* in clot contraction and thrombosis.

## Abstract 2

Genome‐wide association studies link the actin‐binding *Tropomyosin 1* (*Tpm1*) to quantitative blood trait variation, but related mechanisms are unclear. *Tpm1KO* increases platelet lifespan, diminishes adhesion to fibrinogen, dysregulates clot contraction, and accelerates thrombosis. Chemical *Tpm1* inhibition also reduces platelet adhesion and spreading. Our findings define a novel factor (*Tpm1*) governing platelet hemostasis and thrombosis, likely by perturbing platelet mechanosignaling.

## Introduction

Genome‐wide association studies (GWAS) have linked thousands of loci to quantitative blood trait variation, but related genes and mechanisms remain elusive for most sites [1, 2]. Platelet traits, such as platelet count (PLT) and mean platelet volume (MPV), are highly heritable (∼54–87%) [3]. Yet, mechanisms and cell types that are relevant for altered platelet traits are complex. Factors intrinsic to developing blood cells (hematopoiesis), megakaryocytes (megakaryopoiesis), or platelets (thrombopoiesis) can influence platelet formation. Extrinsic factors like obesity or inflammation also influence platelet traits by altering the bone marrow microenvironment or mature platelet lifespan in circulation [4]. Blood cells interact with all organs and impact complex disease states, highlighting a need to reveal mechanisms underlying blood trait variation [5].

We identified the *Tropomyosin 1 (TPM1)* gene locus from blood trait GWAS [6]. Tpm1 encodes an actin‐binding protein that regulates actin cytoskeletal dynamics in many cell types [7]. Actin broadly impacts cell structure, movement, and signaling. *TPM1* deficiency increases the formation of hemogenic endothelial cells (HECs), the embryonic precursors to hematopoietic stem and progenitor cells (HSPCs) that ultimately colonize the bone marrow and support lifelong hematopoiesis [8]. Embryonic lethality from *Tpm1*‐related cardiac dysmorphology prevented assessment of how *Tpm1* knockout impacted postnatal hematopoiesis and blood cell function [8, 9].

The strongest GWAS signals at the *TPM1* gene locus relate to platelet traits (p<10^−100^) [1, 2]. Platelets are critical for hemostasis and inflammation [10]. Blood vessel injury exposes factors that cause platelet activation and adhesion, initiating hemostatic responses [11]. Aggregated platelets form a substrate for fibrin deposition, recruitment of other blood cells, and promote inflammatory responses that augment plug formation and hemostasis. These processes prevent hemorrhage and initiate wound healing, but can also cause thrombotic obstruction to blood flow and risk embolization [11]. Clot contraction is necessary to stabilize clots and permit resumption of blood flow in occluded vessels. Clot formation and contraction demand platelet activation, fibrin binding, and actin‐mediated cytoskeletal remodeling [12, 13]. Therefore, effective hemostasis requires platelet activation, adhesion, and cytoskeletal remodeling – all of which rely on actin filaments to varying degrees.

We hypothesized that *Tpm1* impacts platelet function via actin regulation. We designed this study to ascertain if the effects of *Tpm1* perturbation during embryonic life carry through to postnatal hematopoiesis, or if *Tpm1* has separable effects on platelet function. Using conditional knockout mice and selective chemical inhibition, we reveal novel roles for *Tpm1* in platelet adhesion, clot formation, and thrombosis that are distinct from effects on embryonic hematopoiesis. Our findings advance our understanding of how cytoskeletal regulatory factors impact hemostasis, including a novel place for *Tpm1* among molecules known to impact clot contraction and thrombosis.

## 1 Results

### 1.1 Tpm1 has Separable Roles in Embryonic and Postnatal Hematopoiesis

**FIGURE 1:**
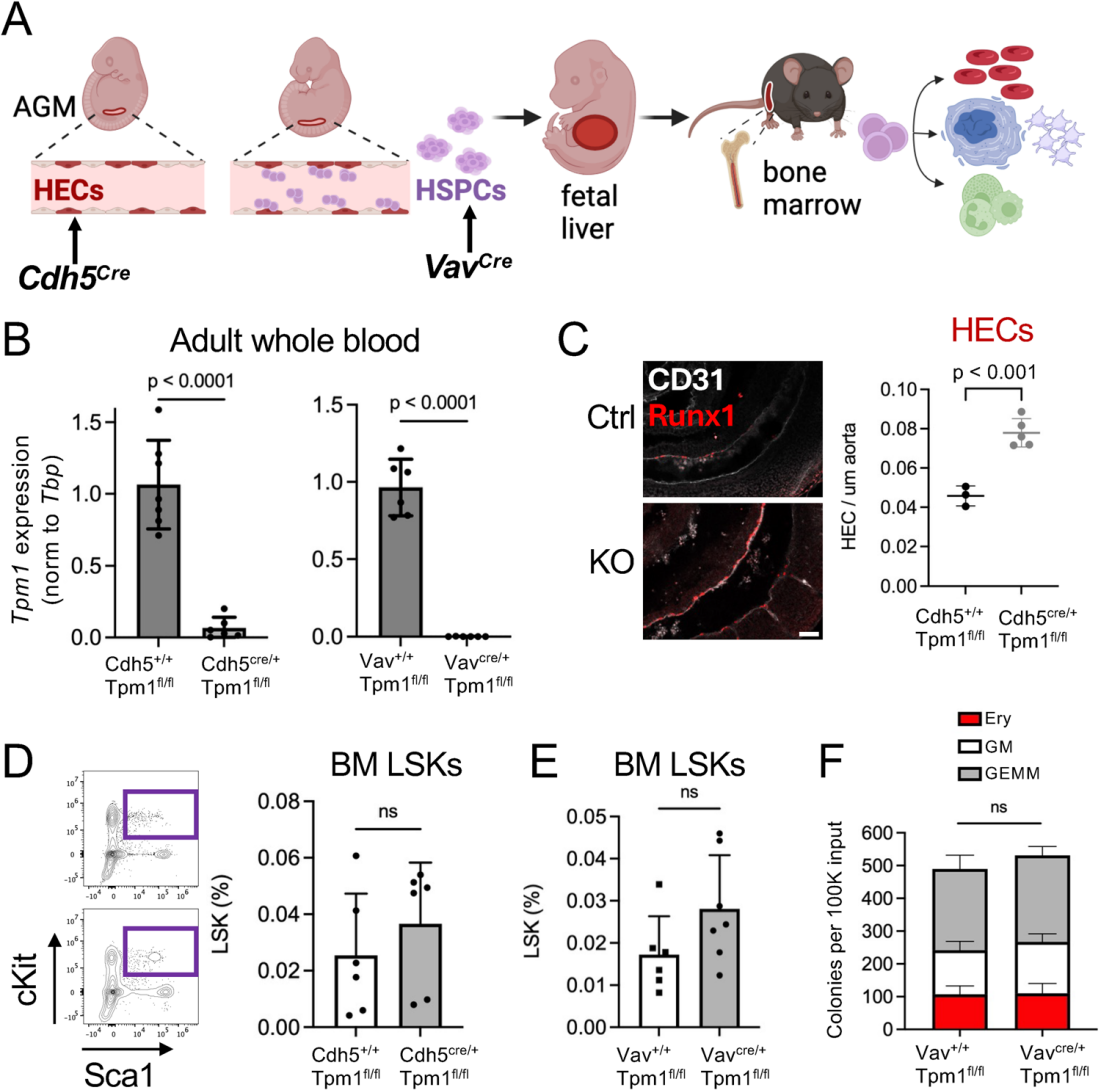
Tropomyosin 1 deficiency impacts formation of hemogenic endothelium but not postnatal bone marrow HSPC quantity or function. (A). Schematic of *Cdh5^Cre^* and *Vav^Cre^* models that are induced at specific stages of developmental hematopoiesis. AGM, aorta‐gonad‐mesonephros region (a major site for definitive HEC production at embryonic day 9.5, E9.5). (B). *Tpm1* mRNA is eliminated in adult peripheral blood cells following conditional embryonic deletion. Semiquantitative real‐time PCR results normalized to *Tbp* (two‐sided t‐test results, n≥6 per genotype). (C). Embryonic *Tpm1* deletion in endothelial cells increases the incidence of Runx1 HECs (two‐sided t‐test result, n = 3‐5 per genotype). (D). Embryonic *Tpm1* deletion with *Cdh5^Cre^* does not increase bone marrow Lin^−^Sca1^+^cKit^+^ (BM LSK) abundance. Representative flow cytometry plot and gating are shown (two‐sided t‐test result, n = 6 per genotype). (E). *Tpm1* deletion in embryonic HSPCs with *Vav^Cre^* does not change bone marrow LSK abundance (two‐sided t test result, n = 6–7 per genotype). (F). Colony formation assays reveal no changes in *Tpm1KO* HSPC function (two‐sided t test performed for each cell type). ns, not significant.

To circumvent cardiac‐related embryonic lethality from *Tpm1* deficiency [8, 9], we generated conditional *Tpm1* knockout (*KO*) models with a floxed *Tpm1* exon 3 (*Tpm1^fl^*, Figure S1A) [14]. Exon 3 is present in all known *Tpm1* isoforms. We crossed *Tpm1^fl^* mice with *Cdh5^Cre^* or *Vav^Cre^* mice to abrogate *Tpm1* expression in endothelial and nascent hematopoietic stem and progenitor cells (HSPCs), respectively [15, 16]. Both models are well validated in the context of developmental hematopoiesis. We used *Cdh5^Cre^*‐mediated deletion to evaluate the effects of *Tpm1* deletion at different stages of developmental hematopoiesis (Figure 1A). The *Cdh5^Cre^* construct is active in HECs [15]. We presumed *Tpm1* deletion in HECs would result in deletion in all HSPCs and peripheral blood cells through adulthood. The *Vav^Cre^* allele can efficiently abrogate gene expression in HSPCs and peripheral blood through postnatal life [16, 17]. We validated efficient recombination and abrogation of *Tpm1* mRNA in peripheral mononuclear blood cells in adult mice compared to littermate controls in both *Cdh5^Cre^* and *Vav^Cre^* models (Figure 1B and Figure S1B).

We further validated by whole mount immunostaining that E9.5 *Cdh5^Cre^ Tpm1^fl/fl^* embryos exhibit increased Runx1^+^ HEC production at E9.5 compared with littermate controls (Figure 1C), similar to *Tpm1‐*deficient mouse embryos [8]. *Cdh5^Cre^ Tpm1^fl/fl^* and *Vav^Cre^ Tpm1^fl/fl^* pups are born at expected Mendelian ratios and exhibit normal viability into adulthood.

We next asked if increased HEC production in *Cdh5^Cre^ Tpm1^fl/fl^* embryos conferred an increase in HSPCs in postnatal bone marrow hematopoiesis (Figure 1A). By flow cytometry, we observe no differences in Lin^−^cKit^+^Sca1^+^ bone marrow HSPCs or peripheral blood counts at steady state (Figure 1D, Figure S2A and Table S1). We similarly find no significant differences in adult bone marrow of *Vav^Cre^ Tpm1^fl/fl^* mice compared to littermate controls, despite previously confirmed Cre activation during fetal life [16] (Figure 1E, F, Figure S2B and Table S2). The lack of change in the postnatal blood progenitors after Tpm1 deletion led us to conclude that the effects of *Tpm1* deficiency during embryonic hematopoiesis are separate from any effects on postnatal hematopoiesis.

### 1.2 Human Genetics Implicates Tpm1 in Mature Platelet Function

**FIGURE 2:**
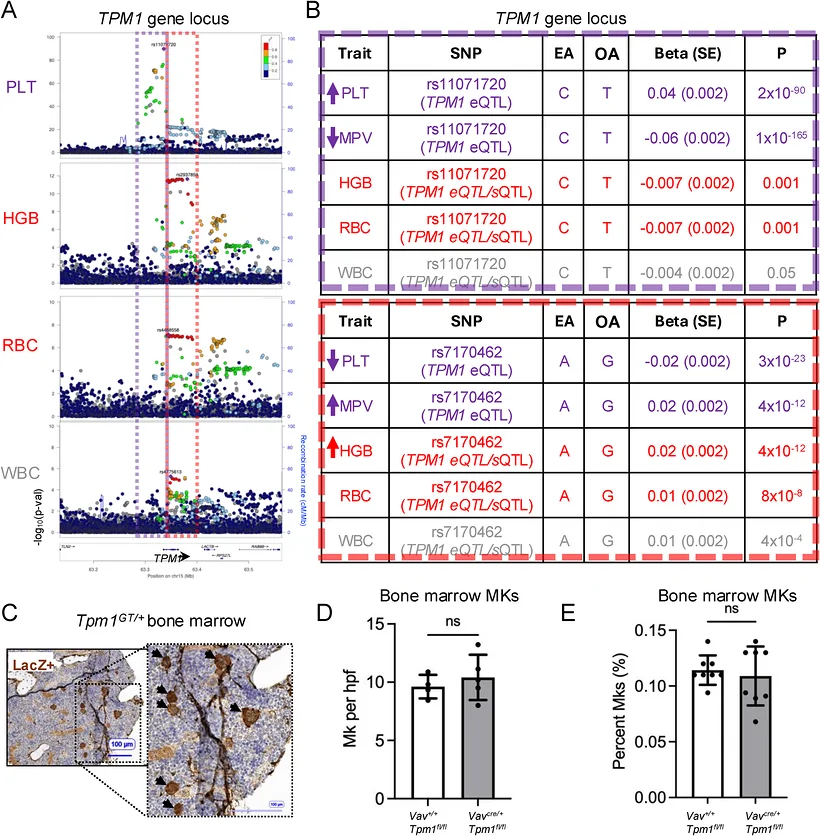
*TPM1* has genetic effects on platelets that are distinct from other blood lineages. (A). Locus zoom plots suggest separate genetic signals for platelet traits and erythroid traits at the *TPM1* gene locus. Purple box indicates loci with statistically significant signals for increased platelet count. The red box indicates loci with significant signals for platelet and erythroid traits. Colocalization analysis points to separate genetic signals for altered platelet count vs erythroid and white blood cell parameters. (B). Key single‐nucleotide polymorphisms (SNPs) at the *TPM1* gene locus that impact blood traits in human GWAS, with representative SNPs taken from the purple and red outlined regions in panel A. SNPs that act as quantitative trait loci for *TPM1* expression (eQTL) and/or splice variation (sQTL) are indicated. Effect alleles (EA) and other alleles (OA) for the indicated directional effects are shown. (C). Immunohistochemistry staining in bone marrow from a *Tpm1^GT/+^* mouse shows strong *LacZ* reporter expression (brown) in megakaryocytes (black arrows). (D). Analysis of sectioned murine bone marrow from *Vav^cre/+^ Tpm1^fl/fl^* vs littermate controls shows no differences in megakaryocyte (Mk) frequency. (E). Flow cytometry analysis of murine bone marrow from *Vav^cre/+^ Tpm1^fl/fl^* vs littermate controls indicates no changes in overall megakaryocyte (Mk) frequency (two‐sided t test results, n = 8 per genotype).

Human genome‐wide association study (GWAS) data have linked single‐nucleotide polymorphisms at the *TPM1* gene locus with quantitative blood trait variation [1, 2]. In light of the disconnect between fetal hematopoietic biology and postnatal bone marrow traits, these GWAS signals suggest that *TPM1* variations affect hematopoietic stem and progenitor cells and/or mature blood cells in circulation. The most statistically significant associations are with platelet traits, including platelet count (PLT) and mean platelet volume (MPV, Figure 2A). However, there are also genome‐wide significant signal associations with hemoglobin (HGB) and sub‐genome‐wide significant signal for variation in red blood cell (RBC) and white blood cell count (WBC, Figure 2A).

Our prior work revealed that systemic factors like obesity can have coordinated impacts on blood traits across lineages, reflecting perturbations in HSPC metabolism and development [4, 5]. In other cases, GWAS signals can have lineage‐specific effects more likely to reflect impacts on mature blood cells in circulation. For example, genetically influenced lipid traits and tobacco use have erythroid‐specific effects that suggest impacts on peripheral erythrocytes [18]. To discern if *TPM1* locus‐related GWAS effects indicated coordinate effects across blood lineages, we performed genetic colocalization analysis [19]. Comparisons of PLT, HGB, and WBC effects reveal platelet‐specific effects at the *TPM1* gene promoter and transcriptional start site that do not colocalize with erythroid or white cell traits (PP4 < 0.02, Figure 2B and Figure S3). These sites include expression quantitative trait loci (eQTL) that overlie relevant hematopoietic transcription factor binding sites and alter *TPM1* expression [6]. Erythroid and white cell traits share colocalized signal within the gene body (PP4 = 0.94, Figure 2B and Figure S3). SNPs in this region include splice quantitative trait loci (sQTL) that alter splicing between *TPM1* exons without altering total *TPM1* mRNA levels [20]. These colocalization findings indicate separate lineage‐specific effects on mature platelets and erythrocytes, or late‐stage precursors for these blood cells, rather than effects on common progenitors for multiple lineages (e.g., HSPCs).

*Tpm1* expression patterns were consistent with a specific role in megakaryocytes and/or platelets. Using a *Tpm1* GeneTrap‐LacZ Reporter model (*Tpm1^GT^*) [8], we confirmed specific robust reporter staining for the reporter construct in bone marrow megakaryocytes (Figure 2C and Figure S4A). However, the overall abundance and morphology of adult bone marrow megakaryocytes were unchanged in *Tpm1^GT^* mice compared to littermate controls (Figure 2D,E and Figure S4B,C).

### 1.3 Tpm1 Impacts Murine Platelet Lifespan and Adhesion Capabilities

**FIGURE 3:**
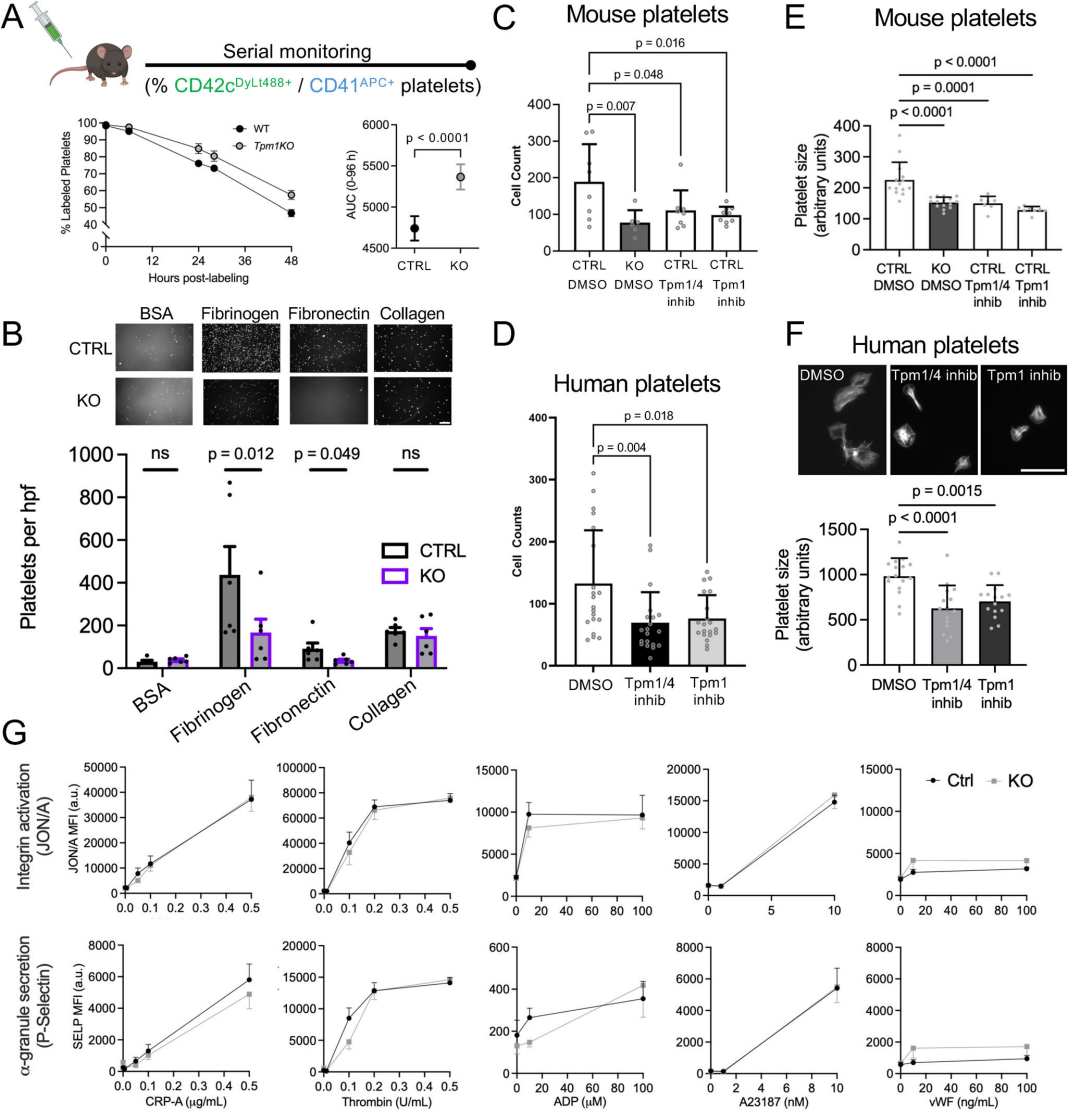
*Tpm1* knockout prolongs platelet lifespan and limits platelet adhesion. (A). Platelet half‐life measurement after labeling indicates a longer time in circulation for *Tpm1KO* platelets. Platelets were labeled with anti‐CD42c^DyLight488^ antibody and the percentage of labeled platelets was measured by flow cytometry over time. (B). Static focal adhesion experiments show decreased *Tpm1KO* platelet adhesion to fibrinogen and fibronectin‐coated cover slips compared to littermate controls, but no significant change in adhesion to type 1 collagen‐coated cover slips. Representative cover slip images depicting actin^+^ platelets (white) are shown above the bar plot. Scale bar, 25 µm (two‐sided t‐test results for each substrate). (C). Selective Tropomyosin inhibition with small molecules [24] reduces murine platelet adhesion to fibrinogen to the level of *Tpm1KO* (ANOVA p‐values are shown). Compound 189‐1 targets Tpm1.8/1.9 and Tpm4.2 isoforms. Compound 189‐3 selectively targets Tpm1.8/1.9 isoforms. (D). Selective Tropomyosin inhibition with small molecules [24] reduces human platelet adhesion to fibrinogen (ANOVA p‐values are shown). (E). *Tpm1KO* or Tpm1 inhibition limits murine platelet spreading on fibrinogen‐coated cover slips (ANOVA p‐values are shown). (F). TPM1 inhibition limits human actin^+^ platelet (white) spreading on fibrinogen‐coated cover slips. Representative platelet images are shown (ANOVA p‐values are shown; scale bar, 10 µm). (G). *Tpm1KO* platelets show minimal changes in activation or degranulation in response to soluble chemical agonists.

We next chose to interrogate *TPM1* effects on platelet biology, given the statistically robust GWAS effects on platelet traits and a lack of functional effects on bone marrow HSPCs or megakaryocytes. SNPs that decrease *TPM1* expression are linked with increased PLT count (Figure 2A). We reasoned that *TPM1* deficiency could either increase platelet formation and/or decrease platelet clearance in circulation. We have previously shown using in vitro models that *TPM1KO* megakaryocyte formation and maturation is normal [6]. Thus, we asked if platelet clearance is decreased in *Vav^Cre^ Tpm1^fl/fl^* mice, which could help explain an increased steady‐state platelet count. To elucidate the lifespan of platelets lacking *Tpm1*, we injected mice with anti‐platelet CD42c^DyLt488^ antibody and monitored the lifespan of labelled CD42c^DyLt488+^/CD41^APC+^ platelets over time by flow cytometry. The mean fluorescence intensity of these platelet surface markers was normal in *Tpm1KO* platelets compared with littermate controls (Figure S5A). *Tpm1KO* mice have longer platelet lifespan compared to littermate controls, as evidenced by an increased area under the curve (5366 ± 153 KO vs 4741 ± 148 Ctrl, p<0.0001) and a ∼50% increased half‐life (86 h vs 58 h in littermate controls by linear regression, Figure 3A and Figure S5B,C). Thus, *Tpm1* normally limits platelet lifespan in murine circulation.

We hypothesized that *Tpm1* deficiency could increase platelet lifespan by limiting clearance from circulation, via limiting adhesion to vascular walls, and/or decreasing activation potential. Focal adhesion biology directly impacts platelet clearance by regulating interactions with vascular walls [21, 22]. Focal adhesions contain nanoscale layers that are functionally specified by tropomyosin isoforms [23]. Tpm1 regulates adhesion maturation and disassembly in these structures [23]. To test *Tpm1KO* platelet adhesion, we compared adhesion of platelets from *Vav^Cre^ Tpm1^fl/fl^* mice vs littermate controls in static cell adhesion assays using fibrinogen, fibronectin, or type I collagen substrates. These substrates directly bind and activate platelets through dedicated platelet membrane receptor protein complexes and can lead to adhesion and activation [22]. After incubating blood with matrix‐coated cover slips for 30 min, we found more platelets retained on all substrate‐coated cover slips compared with bovine serum albumin‐coated controls (Figure 3B and Figure S6A). *Tpm1KO* compromises platelet adhesion to fibrinogen and fibronectin, and to a lesser (non‐significant) effect on collagen (Figure 3B). Importantly, these changes were not due to altered surface receptor abundance (Figure S6B).

We recently used reported small molecule *TPM1* inhibitors to confirm these effects [24]. Murine *Tpm1* inhibition with selective small molecule inhibitors mirrored the effects of *Tpm1KO* on focal adhesion (Figure 3C) and these effects extended to human platelets (Figure 3D). Platelet spreading on fibrinogen was also markedly reduced in the context of *Tpm1KO* or *TPM1* inhibition (Figure 3E,F).

We next evaluated murine platelet activation potential ex vivo using primary murine platelets from *Vav^Cre^ Tpm1^fl/fl^* mice or littermate controls [25]. We measured integrin aIIbb3 activation (CD41^+^JON/A‐PE^+^) and degranulation (CD41^+^P‐Selectin^+^) after incubation with thrombin, collagen‐related peptide A (CRP‐A), adenosine diphosphate (ADP), von Willebrand factor (vWF), and calcium ionophore (A23187). *Tpm1KO* platelets show minimal changes in activation potential across soluble agonists (Figure 3G and Figure S6C). Our findings suggest that platelet *Tpm1KO* activation may be altered in certain contexts, but that *Tpm1* exerts more prominent effects on platelet mechanosignaling, specifically platelet adhesion and spreading.

### 1.4 Tpm1 Deficiency Impairs Clot Contraction

We then sought to determine the functional consequences of altered platelet properties in *Tpm1KO* platelets. Hemostasis involves both clot formation and subsequent contraction, which together produce a stable hemostatic plug that stops bleeding effectively without obstructing blood flow [11, 12, 26]. Clot contraction is driven by activated platelets that adhere to fibrin fibers, forming a three‐dimensional viscoelastic scaffold. One of the downstream effects of platelet activation is the interaction between intracellular actin and non‐muscle Myosin IIA [27].

**FIGURE 4:**
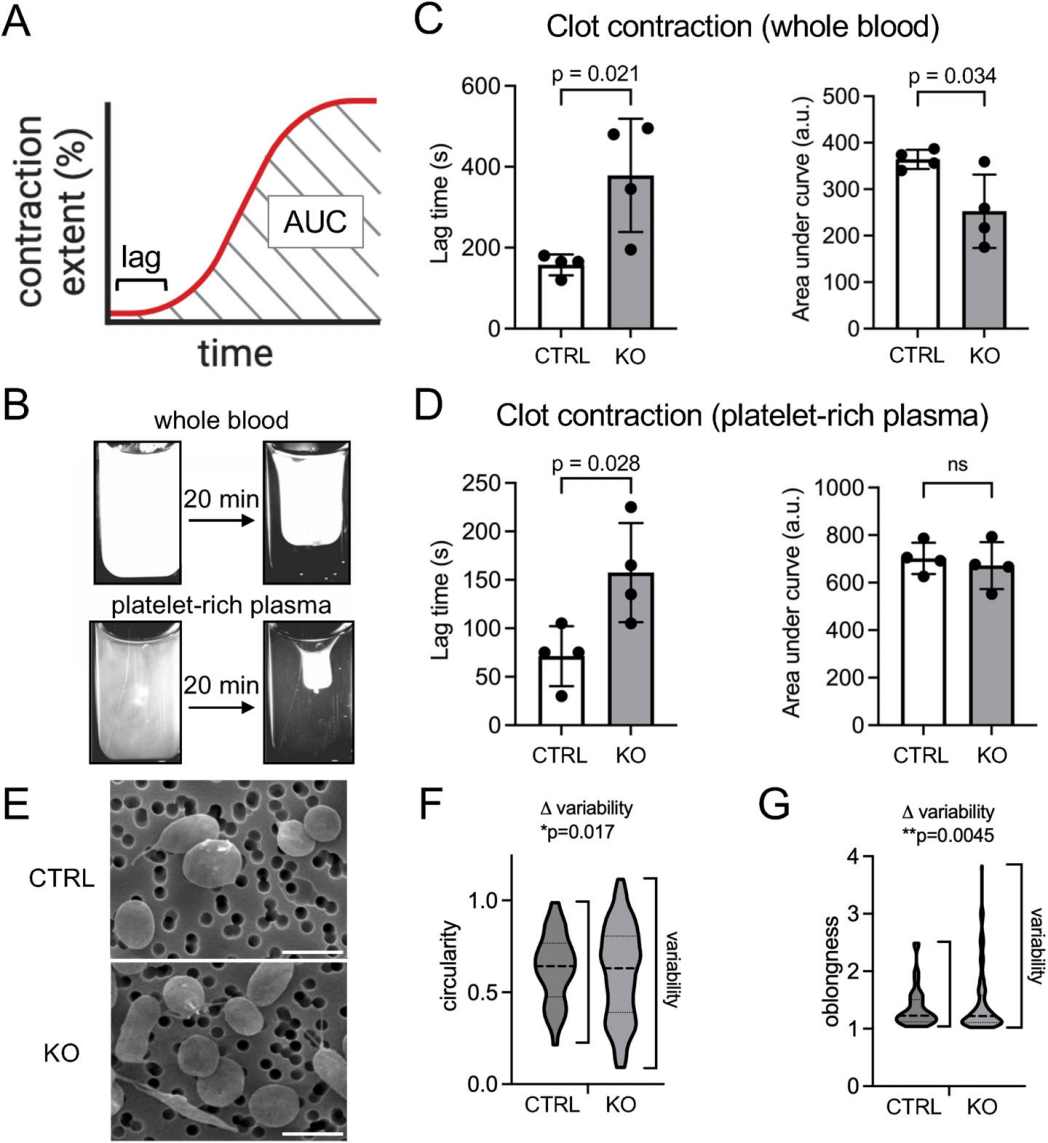
*Tpm1KO* delays initiation of clot contraction. (A). Clot contraction assay schematic depicting measurements of lag time, contraction extent, and total area under the curve (AUC). (B). Exemplary images of whole blood and platelet‐rich plasma before and after clot contraction. (C). Clot contraction initiation is delayed, and the area under the curve for clot contraction is limited in *Tpm1KO* whole blood compared to littermate controls (two‐sided t‐test p‐values). (D). Clot contraction is delayed in *Tpm1KO* platelet‐rich plasma (PRP) compared to littermate controls (two‐sided t‐test p‐values). (E). Representative scanning electron microscopy images of *Tpm1KO* and littermate control platelets. Scale bar, 3 µm. (F). *Tpm1KO* platelets show increased variability in circularity (p‐values represent two‐sample F‐test for equality of variances). (G). *Tpm1KO* platelets show increased variability in oblongness (p‐values represent two‐sample F‐test for equality of variances). ns, not significant.

We tested *Tpm1KO* platelet functionality using a blood clot contraction assay, which quantifies the degree of clot volume reduction as a measure of actomyosin‐dependent platelet contractility [28, 29, 30] (Figure 4A,B). Compared to littermate controls, whole blood samples from *Tpm1KO* mice show a significant delay in the initiation of clot contraction and decreased area under the curve for total clot contraction, while retaining normal contraction velocity and extent of maximal contraction (Figure 4C and Figure S7A,B).

We then determined if these findings were due to platelet‐specific effects. Using platelet‐rich plasma isolated from *Tpm1KO* and littermate controls, we observed a significant delay in the initiation of clot contraction lag time (Figure 4D and Figure S7C,D). These results confirm a functional consequence for abnormal platelet function in *Tpm1* deficiency, which is consistent with adhesion and activation defects and may lead to physiologic implications.

Scanning electron microscopy imaging confirmed altered morphology among some *Tpm1KO* platelets, including elongated resting platelets (Figure 4E). An increased variability in circularity and oblongness was evident in *Tpm1KO* platelets (Figure 4F,G). These findings are consistent with altered platelet actomyosin contractility [27], further suggesting that compromised actomyosin contractility may underlie focal adhesion defects in *Tpm1KO* platelets.

### 1.5 Tpm1KO Accentuates Murine Thrombosis

**FIGURE 5:**
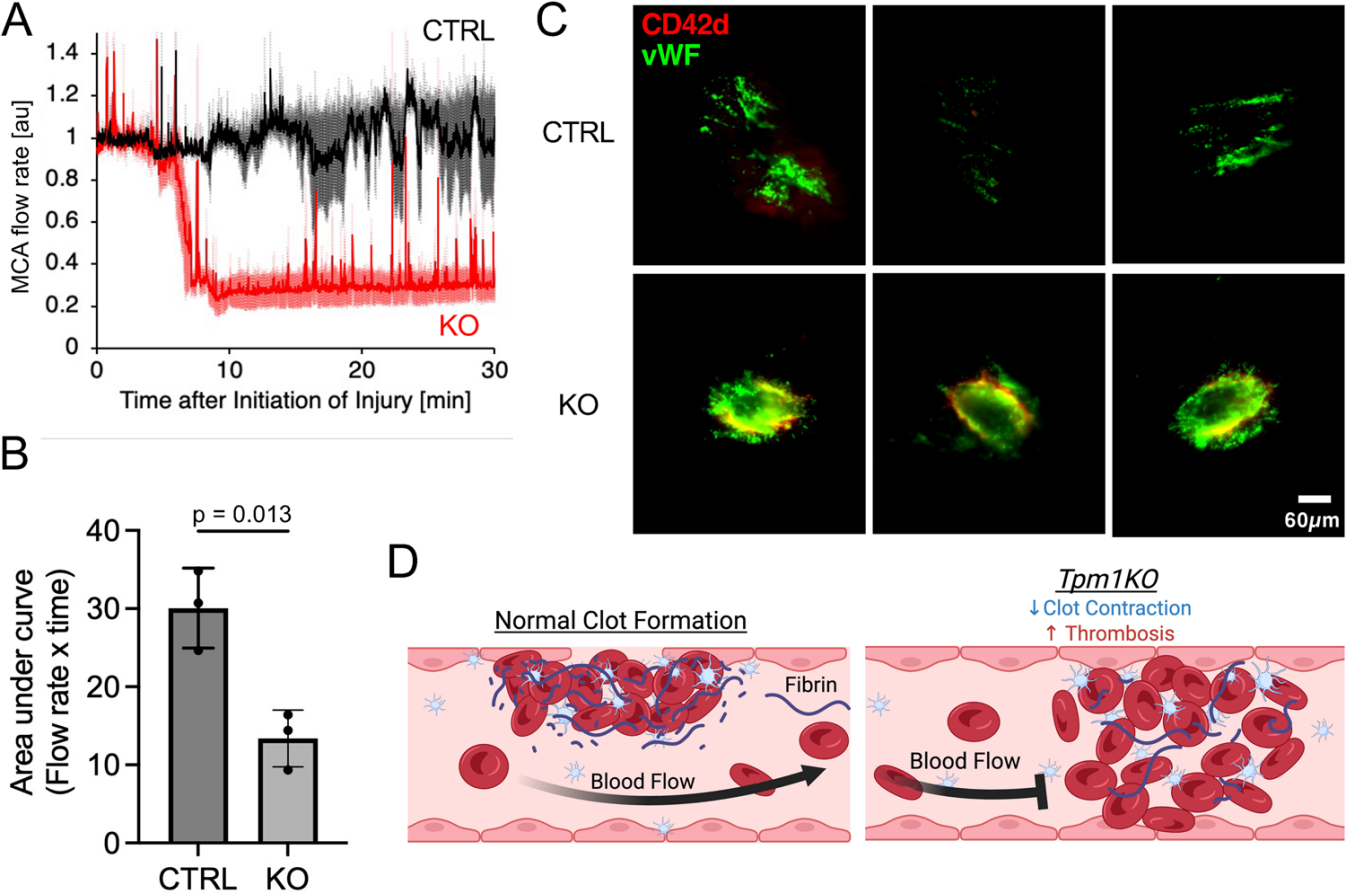
Tpm1KO enhances ferric chloride‐induced middle cerebral artery (MCA) occlusion via enhanced platelet interactions with vWF. (A). Flow rate curves depicting time to MCA occlusion following ferric chloride‐induced injury (n = 3 per genotype). (B). Quantitative changes for MCA occlusion area under the curve (n = 3 per genotype; two‐sided t‐test p‐values). (C). Clot histology showing platelets (CD42d^+^) and vWF aggregation at the site of MCA vessel injury. KO mice showed more circumferential platelet aggregation and vWF accumulation. (D). Schematic for *Tpm1KO* effects on clot contraction and thrombosis.

Platelet focal adhesion is necessary for efficient clot contraction and prevention of pathologic thrombotic extension [28]. These effects are somewhat counterintuitive, since defective focal adhesion or platelet activation can also compromise hemostasis and result in bleeding [31, 32]. To define the hemostatic implications for altered focal adhesion in *Tpm1* deficiency, we tested the effects of *Tpm1KO* in an established murine stroke model [33]. After ferric chloride‐induced injury to the middle cerebral artery (MCA), we monitored blood flow and subsequent clot pathology. Compared to littermate controls, *Tpm1KO* shortens the time to abrogation of blood flow at the injury site (flow rate x time area under the curve [AUC] 30.1 ± 5.1 vs 13.4 ± 3.6, mean±SD, p = 0.01, Figure 5A,B). MCA blood clot histology from control and KO mice confirmed an enhanced interaction of *TPM1KO* platelets with von Willebrand factor (vWF) in the context of vessel injury (Figure 5C). However, we did not detect significant changes in bleeding times nor coagulation pathway activation at steady state in *TPM1KO* mice compared to littermate controls (Figure S8).

These effect directions were reproducible in a common carotid artery (CCA) thrombosis model, with *Tpm1KO* mice showing reduced by non‐significant reductions in time to occlusion and injury site blood flow by AUC (Figure S9A,B). When meta‐analyzed with MCA results, the AUC was significantly decreased in *Tpm1KO* compared to controls (decreased by 45±13%) with moderate heterogeneity across models (I^2^ = 39%, Figure S9C). Interestingly, scanning electron microscopy of early CCA clots from *Tpm1KO* mice showed diminished fibrin networks with minimal branching compared with littermate controls (Figure S9D). These findings show that, in the context of ferric chloride‐induced vessel injury and inflammation, *TPM1KO* platelets have an enhanced response to promote thrombosis but perhaps an impaired ability to bind fibrin during early clot formation. Our results demonstrate a novel functional consequence for *Tpm1KO* on platelet biology and hemostasis (Figure 5D), with potential implications for thromboembolic stroke.

## 2 Discussion

This study reveals novel roles for *TPM1* in platelet adhesion and clot contraction. These effects are distinct from *TPM1* impacts on embryonic hematopoiesis [8] (Figure 1) and are intrinsic to platelets, as opposed to endothelial cells or non‐hematopoietic cell types that support vascular niches during embryonic development [34]. Our findings agree with emerging roles for *TPM1* in mediating focal adhesion stability [23] and actomyosin contractility during clot contraction [11, 12]. To our knowledge, *TPM1* is the first actin regulatory molecule implicated in clot contraction outside of Myosin IIA and fibrin [11, 12]. We anticipate that our findings will lead to further insights into platelet biology and hemostasis, given the extensive prior work on *TPM1* in the context of cardiac sarcomeres [9].

Mammalian actin filament diversity is achieved through expression of 4 genes (*TPM1‐*4) that encode more than 40 isoforms through alternative splicing [7]. Each isoform can impart unique properties on actin filaments. Our murine and iPSC models disrupt all *TPM1* isoforms [6, 8, 35] so we cannot yet parse isoform‐specific *TPM1* functions in platelets. In fact, high molecular weight and low molecular weight *TPM1* isoforms have distinct actin regulatory roles in some cell types [36]. Future isoform‐specific perturbation will parse individual tropomyosin contributions to actin cytoskeletal regulation of platelet functions. In addition, *TPM4* is highly expressed in platelets, and *TPM4* loss causes macrothrombocytopenia (i.e., reducing platelet counts) with a mild effect on platelet function [37]. *TPM1* and *TPM4* may genetically interact to regulate distinct but complementary aspects of platelet biology, which could be elucidated through combined perturbation using genetic models.

Our findings can help explain the GWAS signal at the *TPM1* gene locus [1, 2]. Rather than effects on platelet production [37], we propose that SNPs at the *TPM1* locus extend platelet lifespan to increase steady‐state platelet count. Human exposure to infections, inflammation, and other stressors could amplify functional consequences of altered human platelet lifespan, enhancing the impact of allelic variation that changes *TPM1* expression (Figure 2). The controlled living conditions of our inbred mice may mask phenotypic variation that emerges in real‐world human cohorts with large sample sizes, explaining the lack of variation in platelet count in our mice. Future studies will explore the functional consequences of recurrent infection or inflammation on platelet biology in the context of *Tpm1* deficiency.

Our results are consistent with a role for Tpm1 in platelet actomyosin contractility. Impaired focal adhesion, platelet spreading, clot contraction, and elongated platelet morphology by scanning electron microscopy [27] all suggest defective mechanosignaling in the context of *Tpm1KO* or TPM1 inhibition. Tpm1 may also facilitate platelet adhesion by stabilizing actin filaments at focal adhesion sites, possibly through binding and/or stabilizing Talin and integrin complexes at a nanoscale level [23]. Interestingly, Tpm1 cardiac pathology is ameliorated with concurrent Talin mutation [38]. This genetic interaction in cardiac dysfunction may extend to platelets. Our static adhesion results indicate that *Tpm1* loss perturbs platelet adhesion to fibrinogen and fibronectin, but not type I collagen (Figure 3). Talin facilitates platelet adhesion to fibrinogen and fibronectin, while collagen binding can be Talin‐independent through GPVI [22]. Thus, our findings agree with a molecular role for Tpm1 in conjunction with Talin and/or actomyosin contractility at the platelet cell surface.

Our findings add a novel actin regulatory protein (TPM1) to the small group of molecules known to regulate clot contraction and thrombosis, which also includes fibrinogen, integrins, protease‐activated receptors (PARs), and myosin IIA [11]. Defective clot contraction results in larger, less dense, mechanically fragile thrombi with increased risks of embolization and vascular occlusion [11, 39]. Our findings highlight the role of actomyosin regulation in clot contraction, an established role for *Tpm1* in cardiac muscle [7]. We expect *Tpm1* is relevant in platelets and potentially other cell types (e.g., erythrocytes [40]), since we identified defective clot contraction in assays of *Tpm1KO* whole blood but not PRP (Figure 4). Promoting *Tpm1* activity or otherwise targeting related mechanisms offers a novel translational strategy to prevent or ameliorate thrombosis and stroke pathology (e.g., via gene delivery to hematopoietic stem and progenitor cells [41]), including studies focused on stroke‐related neurological outcomes.

This study supports the potential translational utility linked to defining genetic determinants of platelet and blood trait variation, with potential relevance for human stroke risk given suggested genetic effects (Figure S10). Our findings define a novel factor (*Tpm1*) governing platelet adhesion, clot contraction, and thrombosis. Further work to establish developmental and postnatal roles of *Tpm1* will undoubtedly reveal links between actomyosin contractility, cytoskeletal regulation, and hemostasis.

## 3 Materials and Methods

### 3.1 Mouse Model Derivation and Validation

All mouse experiments were approved by the Children's Hospital Institutional Animal Care and Use Committee (IACUC). The floxed *Tpm1* allele was derived from the EUCOMM *Tpm1* GeneTrap‐Reporter (*Tpm1^GT^*) mouse construct described in our prior publication [8]. We crossed the *Tpm1^GT^* with a *FlpO* recombinase mouse (Jackson Laboratories, strain 011065) to remove the GeneTrap‐Reporter cassette, leaving exon 3 flanked by loxP sites. After confirming accurate recombination by sequencing and backcrossing 3 generations on a C57BL6/J background, we crossed the *Tpm1^fl^* mice with *Cdh5^Cre^* mice [15] or *Vav^Cre^* mice [16] to generate experimental mice for this study. All mice were genotyped by tail snip PCR (Transnetyx). Genotypes were additionally confirmed for experimental mice by PCR of murine whole blood using the following primers:

*Tpm1* proximal loxP Forward: AAGGCGCATAACGATACCAC

*Tpm1* distal loxP Forward: GAGGAGGCCGAGAAGGCTG

*Tpm1* distal loxP Reverse: CACAGGCTGGAGTCCCTGC

### 3.2 Semiquantitative Real‐Time PCR

We confirmed excision of *Tpm1* exon 3 in the context of *Cdh5^Cre^* or *Vav^Cre^* by monitoring *Tpm1* mRNA expression by semiquantitative real‐time PCR on a QuantStudio 5 instrument (Applied Biosystems). We constructed cDNA libraries from whole mouse blood using Qiagen RNeasy kits according to the manufacturer's instructions. We used the following primers to measure total *Tpm1* and *TATA Binding Protein (Tbp)* mRNA expression:

*Tpm1* Forward: CTGGTTGAGGAGGAGTTGGA

*Tpm1* Reverse: ATGTGCTTGGCCTCTTTCAG

*Tbp* Forward: CTCAGTTACAGGTGGCAGCA

*Tbp* Reverse: ACCAACAATCACCAACAGCA

Similar results were obtained using primers designed to specifically measure low molecular weight *Tpm1* mRNA transcripts, which we previously found to be abundant.

### 3.3 Whole Mount Immunohistochemistry

We set up timed matings to generate *Cdh5^Cre^ Tpm1^fl/fl^* embryos and littermate controls. We harvested E9.5 embryos and genotyped tail remnants using the following primers (as also described in the genotyping strategy in Shibata et al. [14]).

*Tpm1* proximal loxP Forward: AAGGCGCATAACGATACCAC

*Tpm1* proximal loxP Reverse: CCGCCTACTGCGACTATAGAGA

We then performed whole‐mount immunostaining and quantifications as previously described [8], using antibodies to detect CD31^+^ endothelial cells (anti‐mouse CD31 MEC13.3, BD Biosciences) and Runx1^+^ hemogenic cells (Runx1 monoclonal antibody EPR3099, Abcam). Secondary antibodies were Goat anti‐Mouse AF488 and Goat anti‐Rat AF555 (Abcam). We collected images using a Leica confocal microscope and manually quantified flat HECs using ImageJ software. We used an anti‐b‐Galactosidase antibody (ab9361, Abcam) to visualize cells with *Tpm1^GT^* reporter *LacZ* expression in murine sternal bone marrow. Slides were processed and scanned using an Aperio ImageScope (Leica). Presented images were obtained directly from these scans. Images of H&E‐stained bone marrow were captured on a Zeiss Primostar 3 microscope with a 40x objective using an Axiocam 208 camera and Zeiss Labscope software.

### 3.4 Bone Marrow Isolation and Analysis

Bone marrow from adult mice aged 2–3 months was isolated, stained, and analyzed as previously described [8, 17]. Megakaryocytes were identified as CD45^+^CD41^+^ bone marrow cells. We analyzed data using FlowJo software (v10, BD Biosciences). MethoCult colony formation assays were conducted per manufacturer specifications (Stem Cell Technologies).

### 3.5 Complete Blood Counts

We collected blood into EDTA‐coated tubes via retro‐orbital collection and obtained total blood counts using a Hemavet V5 instrument (Drew Scientific).

### 3.6 Genetic Colocalization Studies

We collected and analyzed publicly available genome‐wide association study (GWAS) data [1, 2, 42] using established genetic colocalization software [19]. We inferred a PP4 > 0.8 to indicate true colocalization between two traits [5]. We generated LocusZoom plots using a web‐based plotting interface (https://locuszoom.org).

### 3.7 Platelet Half‐Life Experiments

We injected anti‐CD42c^DyLight488^ antibody (Emfret Analytics) into *Vav^Cre^ Tpm^fl/fl^* and littermate controls (*Vav^WT^ Tpm^fl/fl^*) and monitored the percentage of CD41^APC+^ platelets that were also CD42c^DyLight488+^ over time via serial blood collection. We performed flow cytometry on a BD Cytoflex instrument (Beckman Coulter).

### 3.8 Platelet Adhesion and Immunofluorescence Staining

We pre‐treated glass cover slips with fibrinogen, fibronectin, or type I bovine collagen (Stem Cell Technologies) for 2 h and blocked with 3% bovine serum albumin (Sigma–Aldrich) for 2 h. We collected whole blood into 20 units/mL heparin and incubated on cover slips for 30 min. For drug inhibitor experiments, we incubated whole blood with a selective TPM1.8/TPM1.9 inhibitor (189‐3) or a TPM1.8/TPM1.9/TPM4.2 inhibitor (189‐1) at a concentration of 5 mm on cover slips for the indicated times [24]. After washing gently with PBS, we fixed adherent cells in 4% paraformaldehyde, stained with Phalloidin‐AF488 (Invitrogen) per manufacturer's instructions, and mounted on glass slides using ProlongGold with DAPI (Invitrogen). We found that this staining strategy effectively highlighted actin‐rich DAPI‐negative platelets while excluding erythrocytes and rare DAPI^+^ white blood cells. We imaged slides using an Olympus XL microscope and quantified actin^+^ DAPI^neg^ platelets using ImageJ. We quantified the particle size of actin^+^ DAPI^neg^ platelets with ImageJ as a measure of platelet spreading after mouse or human platelets were allowed to adhere and spread on fibrinogen‐coated cover slips for 2 or 4 h, respectively. Human platelet collection was approved by the Children's Hospital of Philadelphia Institutional Review Board (IRB 12–009304).

### 3.9 Platelet Activation Experiments

We conducted platelet activation experiments as previously described [25]. We collected whole blood via retro‐orbital collection into 20 U/mL heparin (Sigma–Aldrich) and activated for 10 min with indicated concentrations of Thrombin (Sigma–Aldrich), Collagen Related Peptide A (CRP‐A, Aniara Diagnostica), calcium ionophore (A23187, Sigma–Aldrich), von Willebrand Factor (vWF, Prolytix/Haematologic Technologies Inc.), or Adenosine 5’‐Phosphate (ADP, Sigma–Aldrich). We measured a_IIb_b_3_ integrin activation (JON/A‐PE^+^, Emfret Analytics) and degranulation (CD62p/P‐Selectin‐FITC^+^, BD Biosciences) among CD41^+^ platelets (CD41‐APC^+^, Thermo Invitrogen) by flow cytometry using a BD Cytoflex instrument (Beckman Coulter). To ascertain platelet surface receptor abundance, we incubated resting platelets with antibodies directed against murine CD41a (MWReg30, eBioscience), CD42b (Emfret), GPVI (JAQ1, Emfret), GPV (Emfret), GP1ba/CD42b (Emfret), or CD49b (eBioscience) and analyzed using a BD Cytoflex instrument (Beckman Coulter). We used FlowJo v10 (BD Biosciences) to analyze data.

### 3.10 Clot Contraction Assay and Measurement

We performed clot contraction assays as previously described [28, 30, 43]. Whole blood was collected directly from the inferior vena cava into syringes containing 3.2% sodium citrate as an anticoagulant at a blood‐to‐anticoagulant ratio of 9:1. For some experiments, platelet‐rich plasma (PRP) was subsequently isolated by centrifugation of whole blood at 200g for 10 min. Citrated mouse blood and PRP samples were activated with 5 U/mL thrombin and 4 mm CaCl_2_, then transferred to transparent plastic cuvettes (7 × 12 × 1 mm) prelubricated with 4% (vol/vol) Triton X‐100 in 150 mm NaCl for whole blood and 1% Pluronic in 150 mM NaCl for PRP to prevent clot adhesion to the cuvette walls. Using light scatter‐based tracking, changes in clot size during contraction were measured every 15 s over a 20‐min period. Serial images of the contracting clot were used to generate a kinetic curve, from which the extent of contraction was determined (defined as the relative reduction in clot size after 20 min).

### 3.11 Murine Thrombosis Models and Histology

We chose to monitor the effects of ferric chloride‐induced injury to the middle cerebral artery (MCA) or common carotid artery (CCA) to assess the role of platelets in established thrombosis models [33]. The MCA was exposed in anesthetized mice and treated with FeCl_3_ (10% w/v, Sigma–Aldrich, applied to the artery via Whatman filter paper saturated with the FeCl_3_ solution). Laser Doppler was used to monitor MCA flow cessation following injury. The MCA clot region was then excised, sectioned, and stained for vWF and CD42d to ascertain clot morphology. Images were obtained on an epifluorescence microscope, and graphical overlays were created using ImageJ. For the CCA thrombosis model, a filter paper saturated with 10% w/v FeCl_3_ solution was applied to the adventitial surface of the isolated artery for 5 min. The CCA was immediately cradled in a perivascular transit‐time ultrasound Doppler flow probe (Transonic) to measure blood flow cessation following injury. At 25 min after application, thrombi were harvested for subsequent analyses.

### 3.12 Electron Microscopy

Resting platelets were collected in sodium citrate anticoagulant, fixed on filter paper, and coated with gold‐palladium (Polaron e5100, Quorum Technologies, UK). CCA thrombi were harvested and immediately fixed in 2% glutaraldehyde in 50 mm sodium cacodylate buffer (pH 7.4) with 100 mm NaCl and incubated at room temperature for 2 h. The samples were then rinsed thrice with the same buffer, dehydrated in a graded ethanol series (30–100 v/v%), immersed in hexamethyldisilazane, and air‐dried. The dry samples were sputter‐coated with gold‐palladium (Polaron e5100, Quorum Technologies, UK). Images of fibrin clots were obtained using a Quanta 250 FEG scanning electron microscope (FEI, Hillsboro, OR).

### 3.13 Tail Bleeding and aPTT Measurement

Tail bleeding experiments were performed as described [33]. Briefly, an incision was made in anesthetized mice, and the tail was submerged in 50 mL of warm PBS. The time to initial cessation of bleeding, total bleeding time, amount of blood lost (as a function of murine weight), and optical density of exsanguinated blood in PBS were quantified. We also measured activated partial thromboplastin clotting time (aPTT) in primary murine blood using standard assays [33].

### 3.14 Statistical Analysis and Graphical Output

Statistics were calculated using GraphPad Prism (v10) or R (v4.4). Graphical outputs used the same software packages. Statistical tests are described within the figure legends, and sample sizes are shown by individual dots within plots. Meta‐analysis was calculated using a random effects model.

## Conflicts of Interest

OAMC is a cofounder of NanoMuse, a startup company developing treatments for stroke and other neuroinflammatory conditions. JWM is a consultant with equity in ChyloMetis, Inc. CST is an inventor on U.S. Patent #12,331,317 (Compositions and Methods for Increasing Megakaryocyte Production) related to *Tropomyosin 1* function during in vitro megakaryopoiesis. ECH and PWG are co‐founders of TroBio Therapeutics, a company focused on developing therapies that target tropomyosins.

## Supporting information




**Supporting File**: advs75930‐sup‐0001‐SuppMat.pdf.

## Data Availability

The data that support the findings of this study are available in the supplementary material of this article.
