# Supplementary material for: Tropomyosin 1 Promotes Platelet Adhesion and Clot Contraction Separate from Its Roles in Developmental Hematopoiesis
Source: Adv Sci (Weinh). 2026 Jun 4;13(48):e17560. doi: 10.1002/advs.202517560 (PMC13336850; doi:10.1002/advs.202517560)
Supplement: Supplementary file 1 [file ADVS-13-e17560-s001.pdf]

Tropomyosin 1 Promotes Platelet Adhesion and Clot Contraction Separate from Its Roles in Developmental Hematopoiesis

Po-Lun Kung[1], Victor Tsao[1], Alina D Peshkova[2], Oscar A. Marcos-Contreras[3,4], Kim Ha[1], Brian M Dulmovits[1], Nkemdilim Okoli[1], Anvi Sinha[1], Gennadiy Fonar[1], Rong Qiu[1], Rolf D Bates[1], Janelle Yeboah[1], Carson Shalaby[1], Tyler Truex[1], Soomin Jeong[3], Edna C Hardeman[5], Peter W Gunning[5], Vladimir R Muzykantov[2,3], Jacob W Myerson[3], Christopher S Thom[1,6,*]

[1] Division of Neonatology, Children's Hospital of Philadelphia, Pennsylvania, PA, USA
[2] Department of Pharmacology, University of Pennsylvania Perelman School of Medicine, Philadelphia, PA, USA
[3] Department of Systems Pharmacology and Translational Therapeutics, University of Pennsylvania Perelman School of Medicine, Philadelphia, PA, USA
[4] Department of Pathology and Laboratory Medicine, Temple University, Philadelphia, PA, USA
[5] School of Biomedical Sciences, University of New South Wales, Sydney, New South Wales, Australia
[6] Department of Pediatrics, University of Pennsylvania Perelman School of Medicine, Philadelphia, PA, USA

*Correspondence
Christopher S Thom
10-052 Colket Translational Research Building
3501 Civic Center Blvd
Philadelphia, PA 19104

Keywords: Tropomyosin 1, platelet, GWAS, actin, hemostasis

Supplementary Information

Supplementary Figures

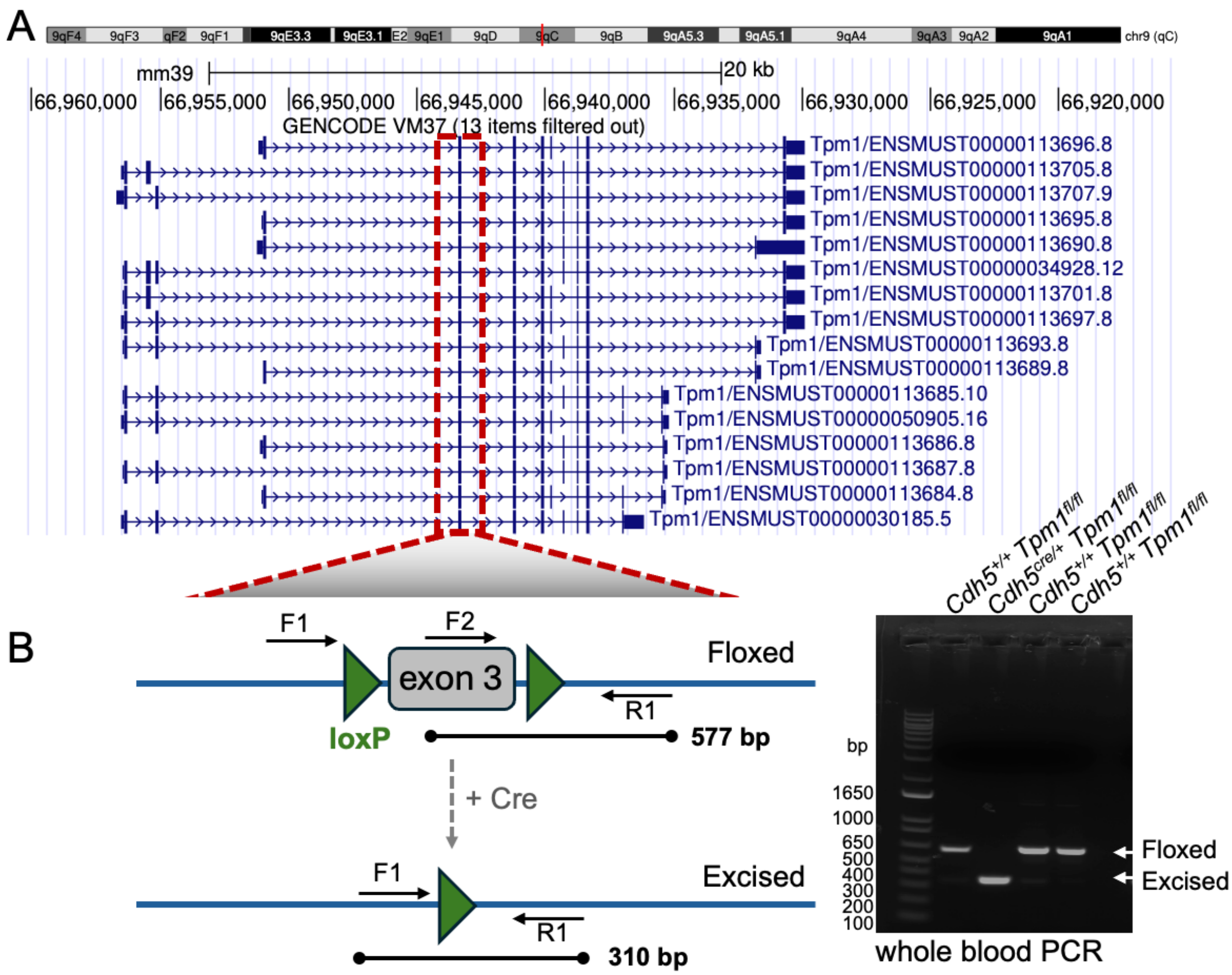

Supplementary Figure 1. Murine *Tpm1* conditional knockout model cassette and confirmation.

A. Murine chromosome 9 ideogram and genome browser track showing the location and transcriptional isoforms produced at the *Tpm1* gene locus. Conditional knockout targets exon 3 (red box), which is part of all known *Tpm1* isoforms.

B. Our murine *Tpm1KO* construct contains loxP sites flanking exon 3. We designed primers (F1, F2, R1) to confirm exon 3 excision following Cre activation. Representative PCR from adult whole blood results show difference between Floxed and Excised alleles. Without excision, primers F1 and R1 would produce a >1300 bp band.

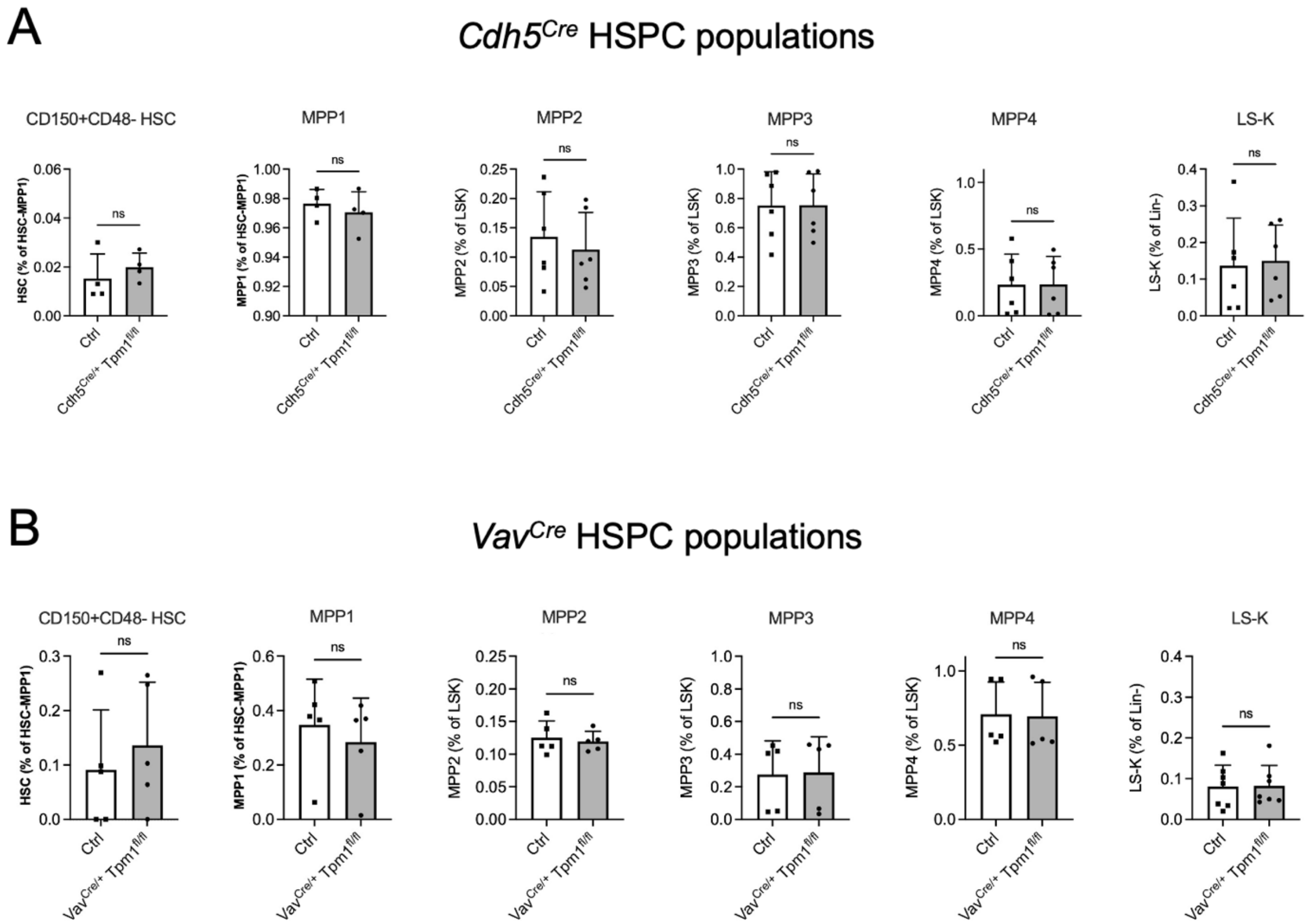

Supplementary Figure 2. Hematopoietic progenitor cell subpopulations for *Tpm1KO* mice and littermate controls.

A. Hematopoietic stem and progenitor cell populations, including $CD150^{+}CD48^{-}$LSK (SLAM HSC) and multipotent progenitor (MPP) cell frequencies, in *Cdh5*$^{cre/+}$ *Tpm1*$^{fl/fl}$ adult mouse bone marrow vs littermate controls.

B. Hematopoietic stem and progenitor cell populations, including $CD150^{+}CD48^{-}$LSK (SLAM HSC) and multipotent progenitor (MPP) cell frequencies, in *Vav*$^{cre/+}$ *Tpm1*$^{fl/fl}$ adult mouse bone marrow vs littermate controls.

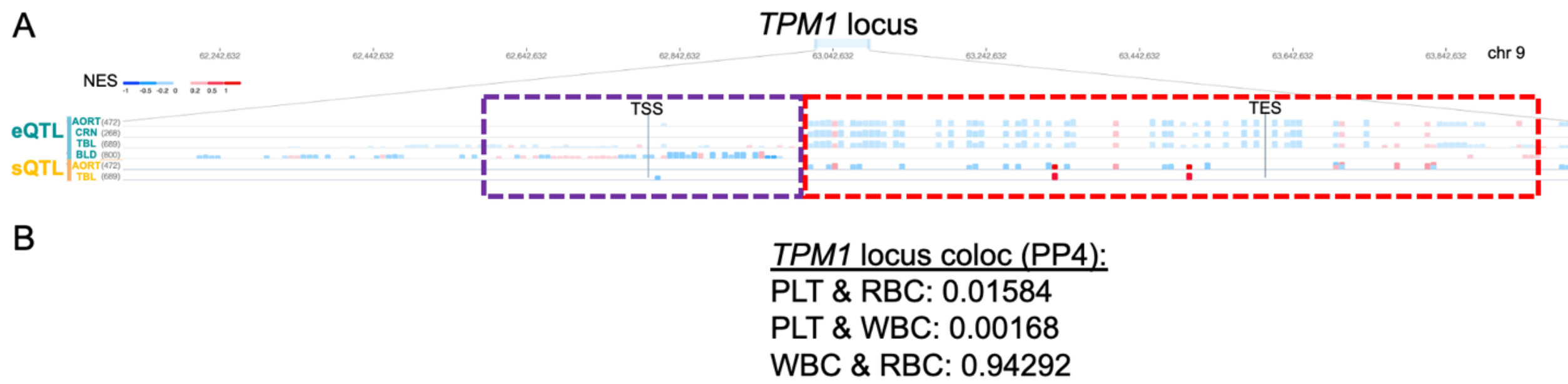

Supplementary Figure 3. Quantitative trait locus map at the *TPM1* locus.

A. Expression quantitative trait loci (**eQTL**) and splice quantitative trait loci (**sQTL**) at the *TPM1* gene locus, as defined by the Gene-Tissue Expression (GTEx) Portal. Individual eQTL or sQTL are shown that impact *TPM1* expression in whole blood (BLD) or blood vessels, including aorta (AORT), coronary (CRN), and tibial (TBL) arteries. There are *TPM1* eQTLs in all cell types, but sQTLs only in aorta and tibial artery. Individual minor allele SNPs that increase (red) or decrease (blue) *TPM1* are indicated by normalized effect sizes (NES). Purple and red boxed areas correspond with areas in Figure 2. Purple region contains predominantly eQTLs in whole blood and specifically affects platelet traits. Red region contains eQTLs and sQTLs and impacts traits across multiple blood cell lineages. TSS, transcription start site. TES, transcription end site.

B. Genetic colocalization results for the indicated traits, centered on rs11071720 (in purple region) but spanning a 250 kb region including the entire *TPM1* locus. A posterior probability for hypothesis 4 (PP4, indicated shared genetic architecture between traits) > 0.8 indicates positive colocalization.

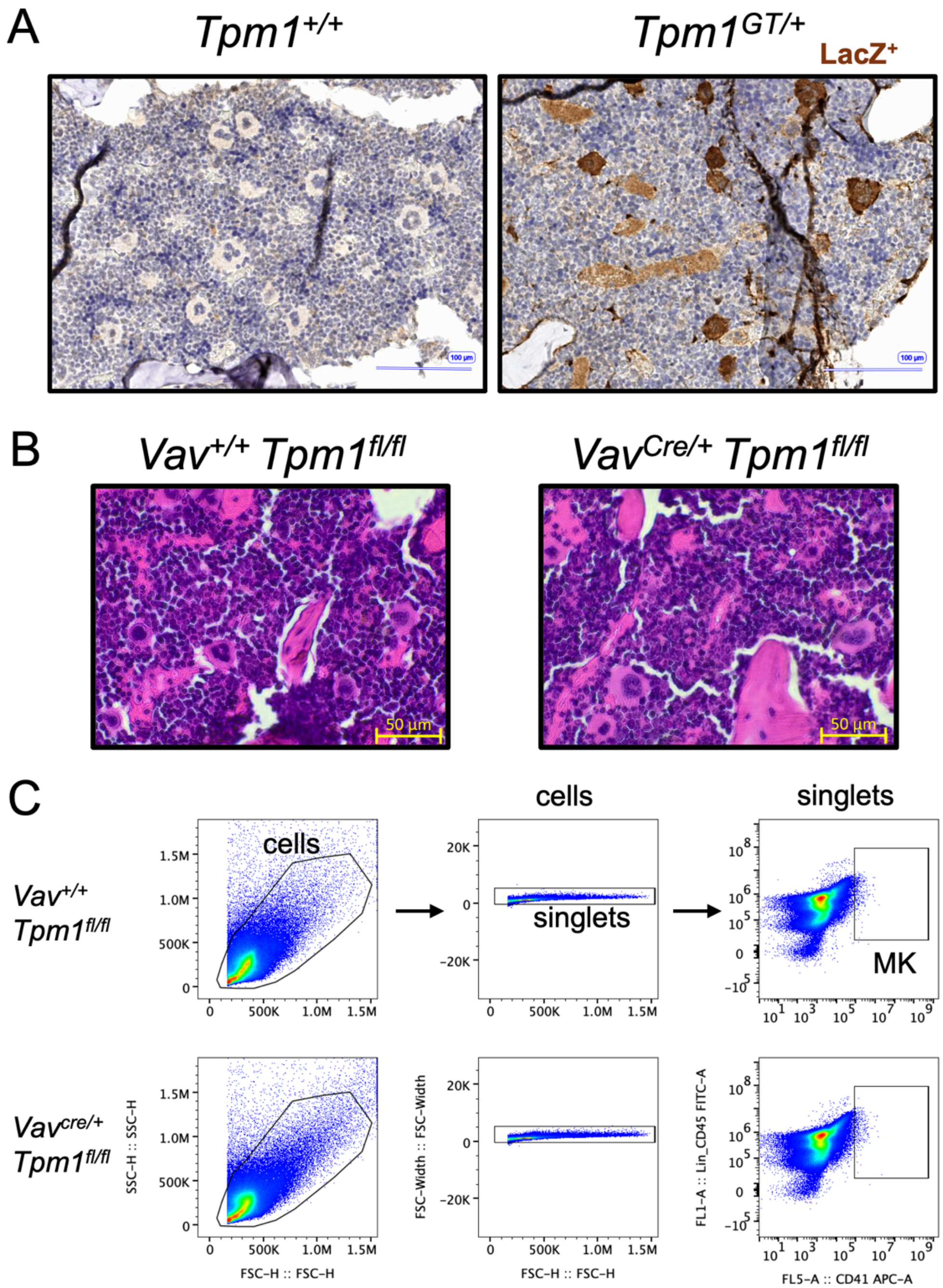

Supplementary Figure 4. *Tpm1* is highly expressed in megakaryocytes but does not affect megakaryocyte quantity in bone marrow.

A. Representative immunohistochemistry images comparing *Tpm1*$^{GT/+}$ sternal bone marrow to littermate controls. Brown staining indicates *Tpm1-GeneTrap-LacZ Reporter* construct activity. Littermate control staining shown for comparison.

B. Representative immunohistochemistry images comparing *Vav*$^{Cre/+}$ *Tpm1*$^{fl/fl}$ femur bone marrow to controls. Large cells with pink cytoplasm are megakaryocytes.

C. Representative flow cytometry plots for megakaryocyte staining in bone marrow.

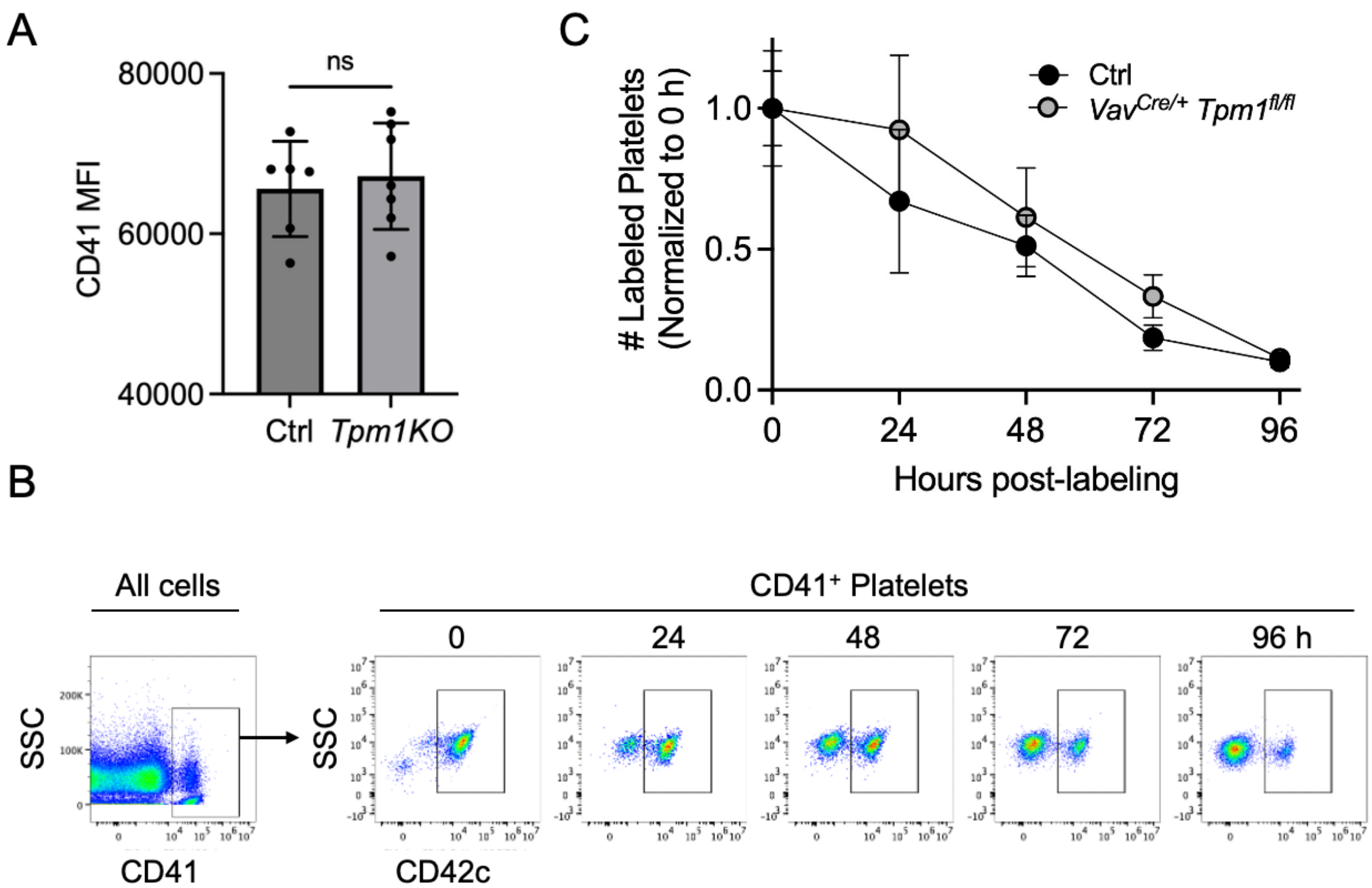

Supplementary Figure 5. ***Vav$^{cre/+}$ Tpm1$^{fl/fl}$*** **platelets have normal CD41 surface expression and prolonged half-life compared to littermate controls.**

A. CD41 abundance is normal in *Vav$^{cre/+}$ Tpm1$^{fl/fl}$* platelets compared to littermate controls.

B. Flow cytometry gating strategy and representative plots for platelet half-life studies.

C. Platelet half-life plots based on absolute CD42c cell counts are similar to analyses based on the percentage of CD41+ platelets, with extended half-life estimates for *Tpm1KO* platelets compared to littermate controls.

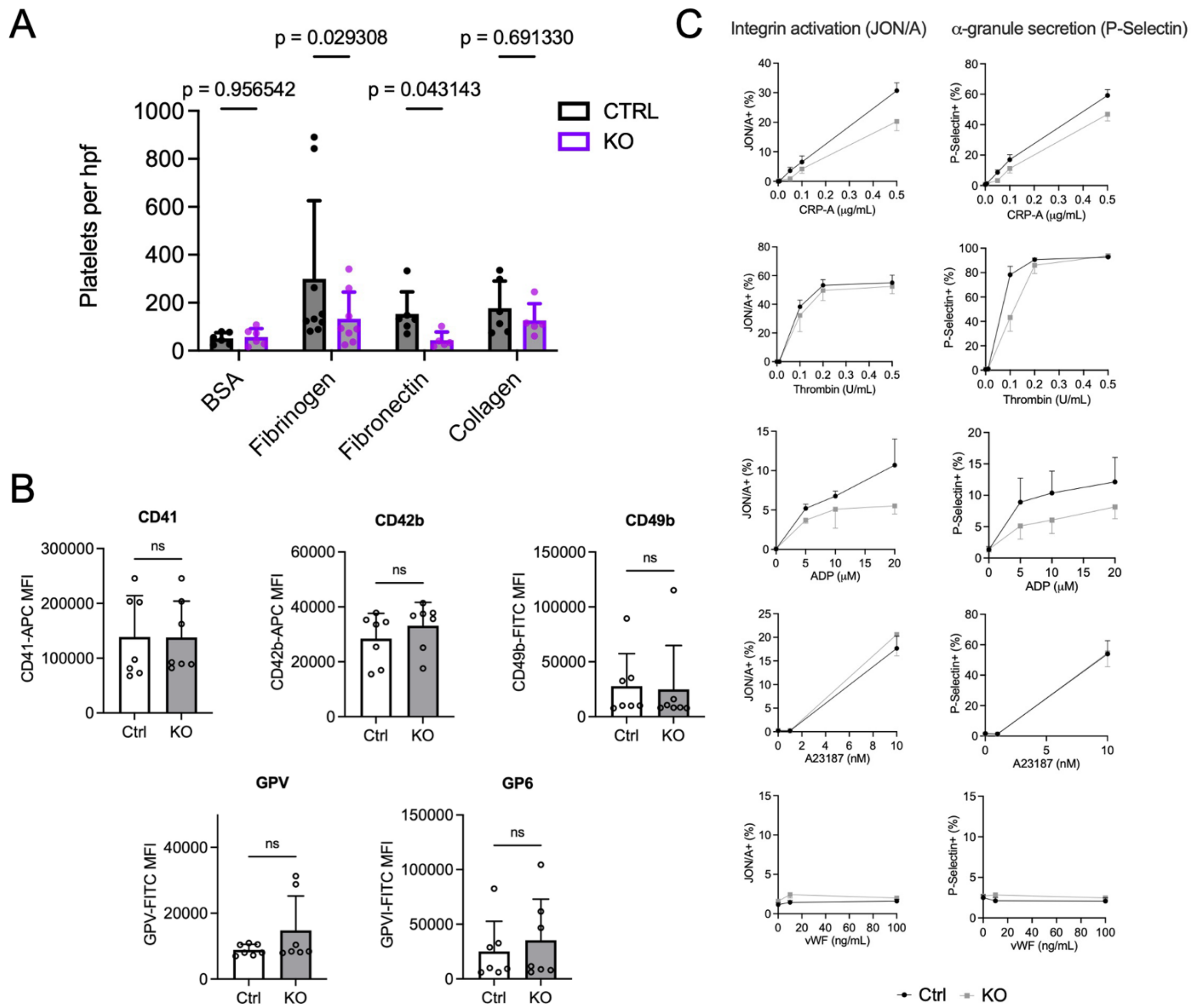

Supplementary Figure 6. *Tpm1KO* prolongs platelet adhesion to select substrates and has variable impacts on platelet activation responses.

A. Static focal adhesion experiments show decreased *Cdh5*$^{cre/+}$ *Tpm1*$^{fl/fl}$ platelet adhesion to Fibrinogen and Fibronectin-coated cover slips compared to littermate controls, but no significant change in adhesion to type 1 collagen-coated cover slips. Significant two-sided t test p-values are shown. ns, not significant.

B. Agonist receptor abundance is unchanged in *Tpm1KO* platelets compared to littermate controls.

C. *Tpm1KO* platelets show variable changes in activation in response to agonists. MFI, mean fluorescence intensity.

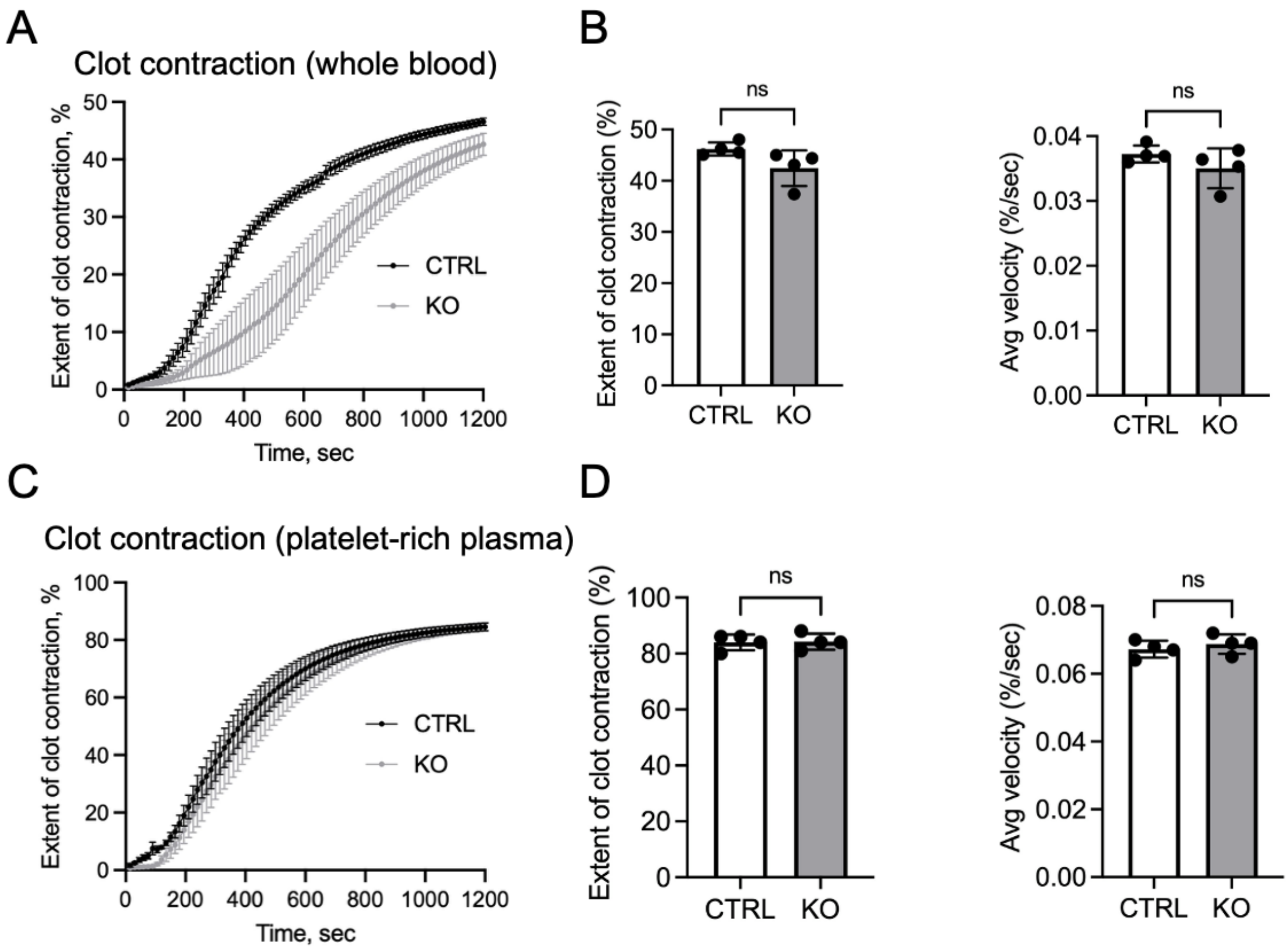

Supplementary Figure 7. Clot contraction parameters for whole blood (WB) and platelet rich plasma (PRP).

A. Whole blood clot contraction curves for *Cdh5*$^{cre/+}$ *Tpm1*$^{fl/fl}$ (KO) vs littermate controls (CTRL).

B. Selected parameters for whole blood clot contraction comparing KO to littermate controls.

C. Platelet rich plasma (PRP) contraction curves for *Cdh5*$^{cre/+}$ *Tpm1*$^{fl/fl}$ (KO) vs littermate controls (CTRL).

D. Selected parameters for PRP clot contraction comparing KO to littermate controls.

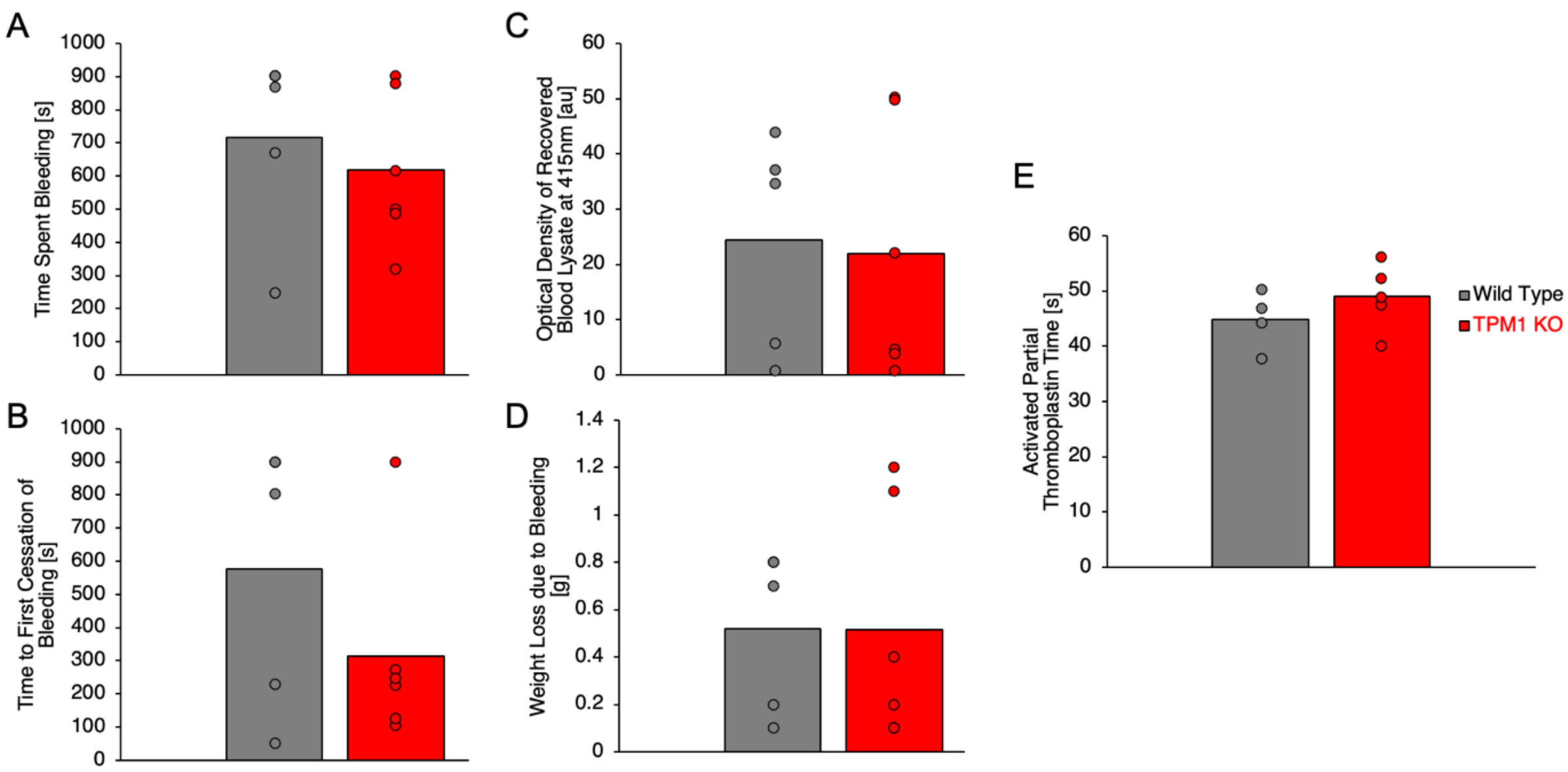

Supplementary Figure 8. Tail bleeding and coagulation parameter measurements for *Tpm1KO* and littermate controls.

A. Time spent bleeding following tail incision.

B. Time to first cessation of bleeding.

C. Optical density of recovered blood following tail bleeding.

D. Weight of blood lost during tail bleeding.

E. Activation partial thromboplastin time. In all plots, Vav^{Cre} $Tpm1^{fl/fl}$ (*Tpm1KO*) are shown in red and littermate controls in gray. No statistically significant changes were detected.

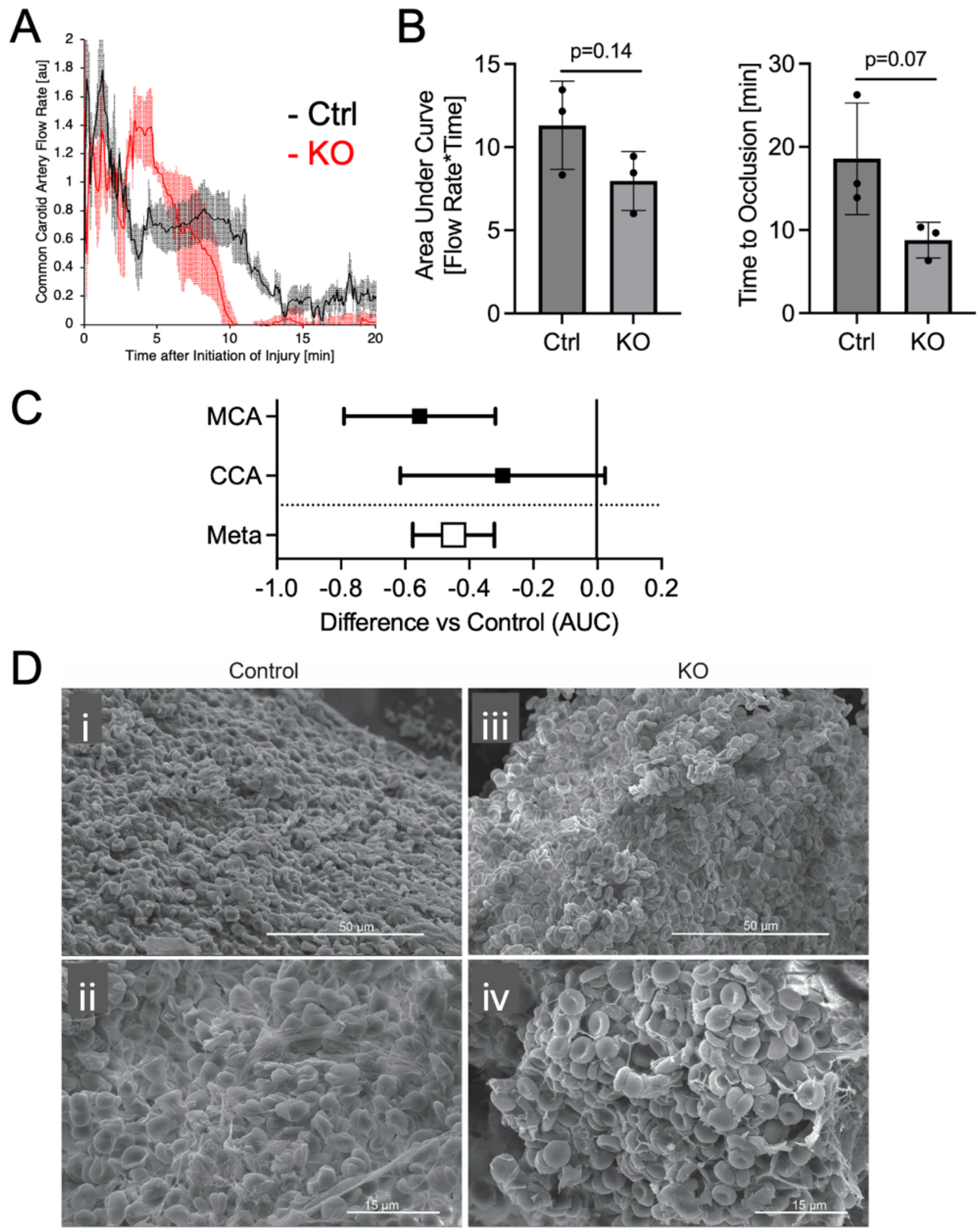

Supplementary Figure 9. *Tpm1KO* effects on clot formation in response to common carotid artery (CCA) thrombosis.

A. CCA flow rate following ferric chloride-induced injury in *Tpm1KO* compared to littermate controls (n=3 per genotype).

B. Comparisons of the area under the curve and time to occlusion following CCA injury in *Tpm1KO* compared to littermate controls (p-values are shown).

C. Meta-analysis forest plot of CCA and MCA results in terms of area under curve (AUC). Box represents mean and whiskers are 95% confidence interval of *Tpm1KO* AUC vs control.

D. Scanning electron microscopy images of CCA thrombi. (i, ii) Control group thrombi exhibit a fibrin network forming a sponge-like structure. Red blood cells (RBCs) are present, with some displaying intermediate forms between erythrocytes and polyhedrocytes. (iii, iv) *Tpm1KO* thrombi show sparse fibrin fibers with minimal branching compared to littermate controls. Scale bars represent 50 μm (i, ii) and 15 μm (iii, iv).

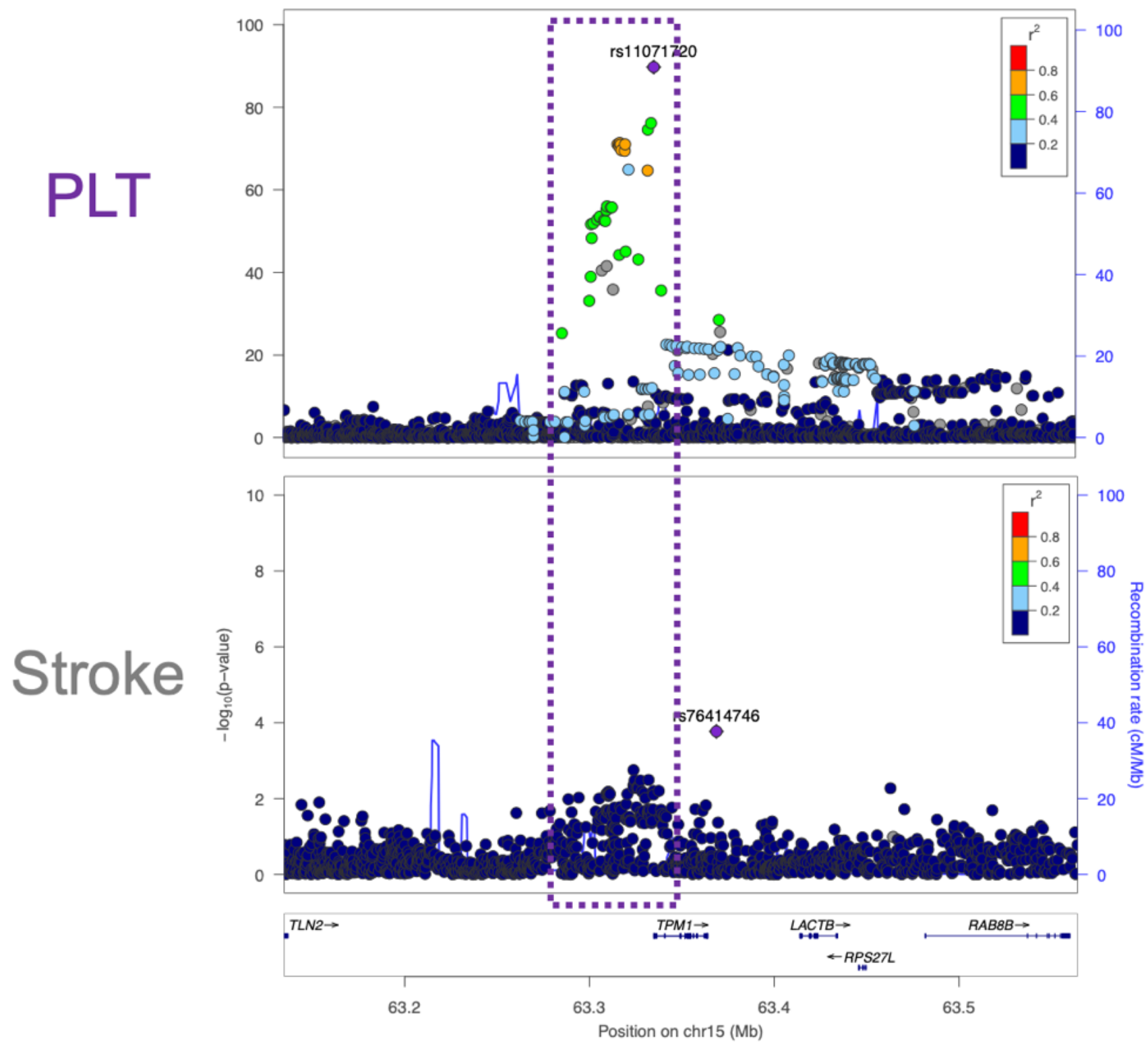

Supplementary Figure 10. Comparison of genetic signals for platelet and stroke.

LocusZoom plots for platelet count (PLT) and stroke. Genetic colocalization results do not predict significant colocalization (PP4 = 0.01) because of the limited signal for stroke at this locus, although we note a region of SNPs with increased stroke risk significance that overlap SNPs that impact platelet count near the *TPM1* gene locus (purple box).

Supplementary Tables

Supplementary Table S1. Blood cell counts from adult *Cdh5*^{Cre} *Tpm1*$^{fl/fl}$ adult mice and matched littermate controls (mean±SD for n=8-10 males per genotype). All values were within normal ranges for adult mice. There were no significant differences between *Cdh5*^{Cre} *Tpm1*$^{fl/fl}$ blood counts and littermate controls. Abbreviations include WBC (white blood cell count), RBC (red blood cell count), HGB (Hemoglobin), HCT (Hematocrit), MCV (mean corpuscular volume), MCH (Mean corpuscular hemoglobin), MCHC (Mean corpuscular hemoglobin concentration), PLT (Platelet count), PCT (platelet-crit), MPV (Mean platelet volume), PDW (Platelet distribution width), RDW (Red cell distribution width), LYM (Lymphocyte count), MON (Monocyte count), NEU (neutrophil count), LY% (Lymphocyte %), MO% (Monocyte %), NE% (Neutrophil %).

Trait (units)	Control	*Cdh5*$^{Cre/+}$ *Tpm1*$^{fl/fl}$
**WBC**	8.23 (2.42)	8.35 (5.08)
**RBC**	8.05 (0.95)	8.40 (0.42)
**HGB**	12.40 (1.51)	13.13 (0.61)
**HCT**	39.06 (5.25)	40.70 (2.15)
**MCV**	56.67 (21.28)	56.83 (21.18)
**MCH**	17.98 (6.11)	18.13 (5.64)
**MCHC**	36.93 (11.69)	37.23 (10.70)
**PLT**	455.83 (54.27)	463.83 (24.90)
**PCT**	0.25 (0.03)	0.29 (0.02)
**MPV**	6.57 (2.37)	7.18 (2.60)
**PDW**	33.92 (11.91)	34.15 (10.66)
**RDW**	26.20 (9.93)	26.77 (9.34)
**LYM**	6.79 (2.81)	6.56 (3.62)
**MON**	0.24 (0.11)	0.20 (0.15)
**NEU**	1.20 (0.77)	1.59 (1.43)
**LY%**	95.63 (46.88)	94.43 (39.49)
**MO%**	3.73 (2.17)	2.83 (1.19)
**NE%**	17.32 (11.70)	19.40 (5.64)

Supplementary Table S2. Blood cell counts from adult *Vav*^{Cre} *Tpm1*$^{fl/fl}$ adult mice and matched littermate controls (mean±SD for n=6 males per genotype). All values were within normal ranges for adult mice. There were no significant differences between *Vav*^{Cre} *Tpm1*$^{fl/fl}$ blood counts and littermate controls. Abbreviations include WBC (white blood cell count), RBC (red blood cell count), HGB (Hemoglobin), HCT (Hematocrit), MCV (mean corpuscular volume), MCH (Mean corpuscular hemoglobin), MCHC (Mean corpuscular hemoglobin concentration), PLT (Platelet count), PCT (platelet-crit), MPV (Mean platelet volume), PDW (Platelet distribution width), RDW (Red cell distribution width), LYM (Lymphocyte count), MON (Monocyte count), NEU (neutrophil count), LY% (Lymphocyte %), MO% (Monocyte %), NE% (Neutrophil %).

Trait (units)	Control	*Vav*$^{Cre/+}$ *Tpm1*$^{fl/fl}$
**WBC**	7.23 (1.74)	5.84 (1.49)
**RBC**	8.13 (0.46)	8.05 (0.78)
**HGB**	13.46 (0.65)	12.94 (1.10)
**HCT**	40.58 (1.93)	40.32 (3.52)
**MCV**	50.00 (1.56)	50.38 (2.20)
**MCH**	16.60 (1.33)	16.09 (0.54)
**MCHC**	33.24 (2.68)	32.08 (0.75)
**PLT**	514.00 (72.99)	536.57 (86.47)
**PCT**	0.38 (0.17)	0.32 (0.07)
**MPV**	5.90 (0.36)	6.14 (0.40)
**PDW**	29.28 (0.92)	30.23 (0.66)
**RDW**	22.79 (4.24)	24.13 (4.28)
**LYM**	6.06 (1.41)	4.92 (1.35)
**MON**	0.15 (0.10)	0.17 (0.06)
**NEU**	1.03 (0.45)	0.75 (0.26)
**LY%**	83.92 (3.35)	84.09 (3.48)
**MO%**	2.22 (1.45)	3.00 (1.12)
**NE%**	13.85 (3.81)	12.89 (3.18)
